# Involvement of Innate Immune System in Late Stages of Inherited Photoreceptor Degeneration

**DOI:** 10.1038/s41598-017-18236-7

**Published:** 2017-12-20

**Authors:** Raghavi Sudharsan, Daniel P. Beiting, Gustavo D. Aguirre, William A. Beltran

**Affiliations:** 1 Division of Experimental Retinal Therapies, Department of Clinical Sciences and Advanced Medicine, School of Veterinary Medicine, University of Pennsylvania, Philadelphia, PA 19104 USA; 2 Department of Pathobiology, School of Veterinary Medicine, University of Pennsylvania, Philadelphia, PA 19104 USA

## Abstract

Retinitis pigmentosa (RP) is a group of inherited retinal degenerations that lead to progressive vision loss. Many mutations in 60 different genes have been shown to cause RP. Given the diversity of genes and mutations that cause RP, corrective gene therapy approaches currently in development may prove both time-consuming and cost-prohibitive for treatment of all forms of RP. An alternative approach is to find common biological pathways that cause retinal degeneration in various forms of RP, and identify new molecular targets. With this goal, we analyzed the retinal transcriptome of two non-allelic forms of RP in dogs, rcd1 and xlpra2, at clinically relevant advanced stages of the two diseases. Both diseases showed very similar trends in changes in gene expression compared to control normal dogs. Pathway analysis revealed upregulation of various components of the innate immune system in both diseases, including inflammasome and complement pathways. Our results show that the retinal transcriptome at advanced stages of RP is very similar to that of other retinal degenerative diseases such as age-related macular degeneration and diabetic retinopathy. Thus, drugs and therapeutics already in development for targeting these retinopathies may also prove useful for the treatment of many forms of RP.

## Introduction

Retinitis pigmentosa (RP) is a heterogeneous group of inherited retinal degenerations that leads to progressive vision loss due to death of rod and cone photoreceptor cells. With a prevalence of 1:4000, RP affects over 100,000 individuals in the US and over a million worldwide^1,2^. Mutations in at least 60 different genes can cause RP (Retnet: https://sph.uth.edu/Retnet/; April 2017). Although there is considerable variation between the various forms of RP, rods are typically affected earlier in the course of the disease and cone impairment follows later. This temporal pattern typically causes night blindness in childhood/adolescence followed by progressive constriction of the visual field leading to retention of only central vision in adulthood. Complete blindness may occur later in life.

There is currently no cure for inherited retinal degenerations. Promising gene therapy approaches have been achieved in both small and large animal models, and some are now being translated towards clinical trials^3^. However, given the high cost to develop a marketable gene-specific therapy that by definition aims at replacing or correcting only one single defective gene, it is very unlikely that this approach will be used to target all the various forms of RP, as several fall within the group of ultra-orphan diseases. Identifying, instead, neuroprotective agents or cell death inhibitors that target cell signaling pathways common to various retinal degenerative diseases, may provide a “pan-RP” therapeutic option. While most proof of concept studies in animals are typically designed to demonstrate efficacy in preventing disease onset or halting retinal degeneration early in the disease, there is also a need for preclinical screening and assessment of such pharmacological compounds at later disease stages. Indeed, progressive retinal degeneration may go undetected earlier in life in asymptomatic RP patients until a significant number of photoreceptors are lost^1^. Therefore, identifying the survival and degenerative cell signaling pathways that are active in retinas at advanced stages of RP may provide relevant molecular targets for developing novel therapeutics.

In order to identify these common biological pathways, we have analyzed at advanced stages of disease (defined as more than 50% loss of photoreceptors) the retinal transcriptomic profile of two naturally-occurring, non-allelic forms of photoreceptor degeneration in dogs, namely, rcd1 and xlpra2. Rcd1 (rod cone dysplasia type 1) is caused by a nonsense mutation in the beta subunit of rod-specific cGMP phosphodiesterase *Pde6b*^4,5^. Xlpra2 (X-linked progressive retinal atrophy 2) is caused by a two-nucleotide deletion and frameshift in exon ORF15 of the retinitis pigmentosa GTPase regulator (*RPGR*) gene that is expressed in both rods and cones^6^. Mutations in both genes cause early onset retinal degeneration in people and in dogs^7–9^. In the canine models, an early burst of photoreceptor cell death is followed by a phase of protracted cell loss over the course of several months^9,10^. This kinetics of photoreceptor cell loss provide a reasonable experimental time-frame to examine the retinal alterations that occur at advanced stages of disease.

Our study shows that the retinas of these two non-allelic canine models of RP shared similar changes in gene expression at late stage disease. Biological pathways related to the innate immune system and incriminated in other retinal degenerative diseases such as age-related macular degeneration (AMD) and diabetic retinopathy (DR), were also found to be activated in both models.

## Results

### Similar transcriptomic profile in rcd1 and xlpra2 retinas at late stage disease

**Figure 1:**
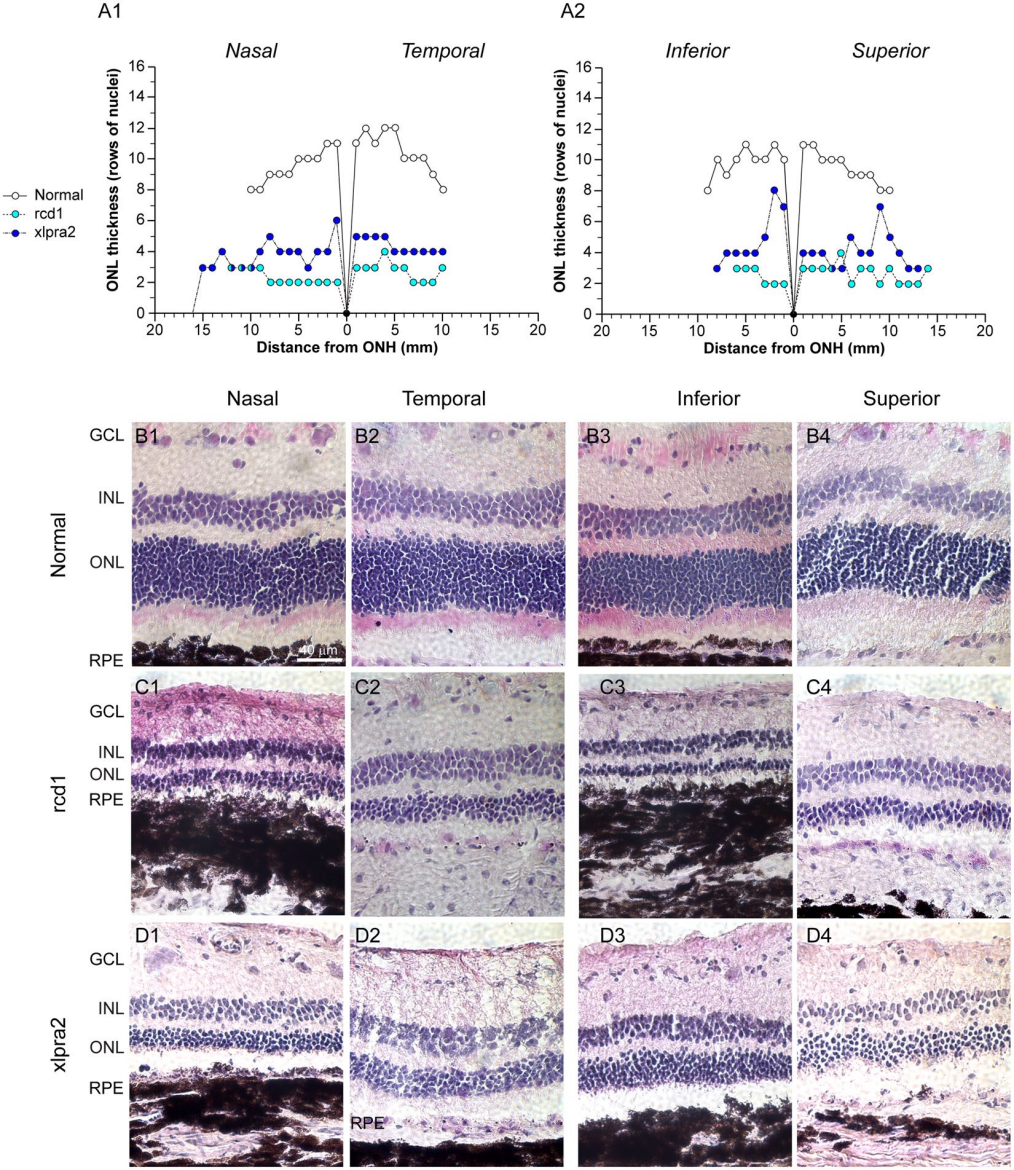
ONL count in normal and mutant retinas. (**A1–2**) Biaxial spidergraphs of outer nuclear layer thickness in the nasal-temporal and superior-inferior meridians for normal, rcd1 and xlpra2 retinas. H&E stained photomicrographs illustrating ONL thickness differences in the nasal, temporal, superior and inferior meridians of retina in a (**B1–4**) normal dog, (**C1–4**) rcd1 dog, and (**D1–4**) xlpra2 dog at 3000 ± 500 microns from the optic nerve head. RPE: Retinal Pigment Epithelium; ONL: Outer Nuclear Layer; INL: Inner Nuclear Layer; GCL: Ganglion Cell Layer; ONH: Optic Nerve Head.

To confirm the advanced stage of retinal degeneration in the three rcd1 (22 weeks of age) and three xlpra2 dogs (41 weeks of age) selected for transcriptomic analysis, their right eye was processed for histological evaluation (Supplementary Table S1). Quantification on H&E stained retinal cryosections of the thickness of the outer nuclear layer (ONL), which contains the photoreceptor somatas, showed a pan-retinal loss of photoreceptors in both diseases (Fig. 1A1–2). The ONL thickness was reduced to less than 50% of normal in all four retinal quadrants (Fig. 1B1–4,C1–4,D1–4). The extent of photoreceptor cell loss was slightly higher for rcd1 compared to xlpra2 consistent with the earlier onset and more severe progression of degeneration in this model^10^. Mean ONL thickness (±S.D.) (measured as rows of nuclei) across all four quadrants was 2.6 (±0.6) for rcd1, 4.2 (±1.1) for xlpra2, and 9.9 (±1.1) for normal dogs. Accurate characterization of the structure of photoreceptor inner and outer segments could not be performed as these retinas were embedded without prior fixation.

**Figure 2:**
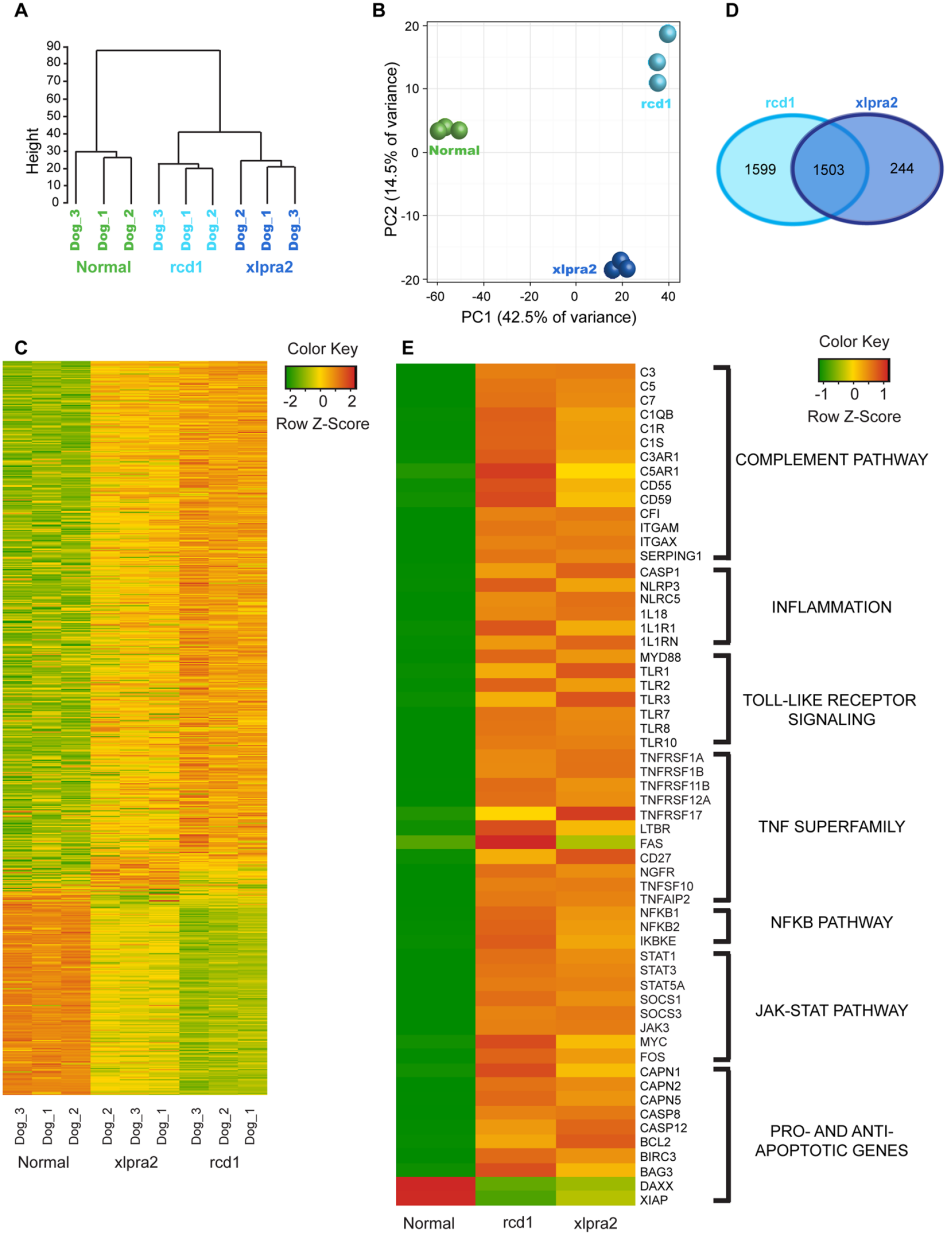
Gene level exploratory analysis and heat maps of differentially expressed genes in mutant retinas. (**A**) Hierarchical clustering of the Euclidean distances on log-transformed expression values in the RNA-seq dataset. (**B**) Principle component analysis on normalized gene expression values in RNA-seq data. (**C**) Heatmap of all differentially expressed genes with a p-value ≤ 0.05 in either one of the two mutants. Green indicates decrease and red indicates increase in gene expression. (**D**) Venn diagram indicating the number of distinct and overlapping differentially expressed genes in the two diseases. (**E**) Heatmap of a subset of differentially expressed genes grouped into pathways related to innate immune system or pro- and anti-apoptotic signaling and molecules.

In order to identify the molecular pathways active at this late stage of these two non-allelic retinal degenerative diseases, we performed RNA-seq analysis on the left neuroretinas from the three rcd1 and xlpra2 dogs, and compared their transcriptomic profile to that of three adult normal dogs. The degree of similarity between the samples was visualized using a hierarchical clustering dendogram that showed distinct segregation of the group of normal dogs from the two sets of mutant animals (Fig. 2A). Each of the three rcd1 and xlpra2 animals further segregated from each other into distinct groups, indicating differences between the late stages of these two diseases. Likewise, a principal component analysis was performed and the first principal component (PC1) separated mutant dogs from the normal dogs, accounting for 42.5% of the variability in the dataset. The second principal component (PC2) separated the two diseases with 14.5% variability in the dataset (Fig. 2B). Thus, initial data assessment showed clear segregation between the three groups of animals, established distinction between normal versus mutant dogs, and indicated commonalities between the two mutant dogs which we explored further by identifying the differentially expressed genes in the dataset.

A comparison between normal and rcd1 animals yielded 8,592 differentially expressed genes (DEGs; FDR ≤ 0.05). xlpra2 samples had 7,031 DEGs (FDR ≤ 0.05) (Supplementary Table S2). A heatmap of all DEGs showed that the levels of expression of a number of genes in the mutant animals was distinct from normal dogs, but that the pattern of retinal gene expression in the two diseases was very similar (Fig. 2C). Of the total DEGs identified, 3,102 genes in rcd1 and 1,747 genes in xlpra2 had a fold change value ≥2. Of the 1,747 DEGs identified in xlpra2 dogs, 1,503 (86%) were in common with rcd1 (Fig. 2D). In summary, our data indicated a high level of similarity in gene expression changes in the mutant retinas at late stages of photoreceptor degeneration.

### Genes involved in innate immune responses are upregulated in late stages of retinal degeneration

*In silico* identification of biological pathways that are differentially regulated in late stage retinal degeneration was performed using the Ingenuity Pathway Analysis (IPA; Ingenuity® Systems, www.ingenuity.com). Only DEGs with FDR ≤ 0.05 and absolute fold change ≥2 or more were used for functional analysis. In order to identify pathways involved in retinal degeneration, only canonical pathways known to be involved in cell signaling, neurodegeneration, and pro-/anti-apoptotic signaling were included in the analysis from the Ingenuity Knowledge Base, whereas most of the metabolic and cancer-related pathways were excluded. IPA identified 144 canonical pathways for rcd1 and 131 pathways for xlpra2 with a p-value of 0.05 or lower (−log(p-value) ≥1.3). Most of these IPA pathways have considerable overlap and redundant DEGs. Of these, 116 canonical pathways were common between the two diseases (Supplementary Table S3). Pathways that were identified in only one disease were also present for the other but did not meet the p-value cutoff. The only pathway unique to rcd1 was GABA receptor signaling. A complete analysis using all canonical pathways in the Ingenuity Canonical Pathway Knowledge Base is provided in Supplementary Table S4.

A number of biological pathways have been previously described by other groups to influence the course of degeneration in various retinal degenerative diseases such as RP, AMD and diabetic retinopathy (DR). These include the complement pathway^11,12^, inflammasome^13,14^, Toll-like receptor (TLR) pathway^15,16^, Tumor necrosis Factor (TNF)^10,17^ pathway, JAK-STAT pathway^18^, and number of pro-survival and cell death pathways^19–22^. The latter include caspase-dependent and caspase-independent apoptotic pathways as well as non-apoptotic cell death mechanisms involving histone deacetylases and poly-ADP-ribose-polymerases^23–29^. Many genes associated with these previously described pathways were found to be upregulated in the transcriptome of rcd1 and xlpra2 retinas at late stages of the diseases (Fig. 2D, Table 1, Supplementary Table S2). Curating the IPA data pointed towards the involvement of multiple innate immune responses and illustrated the complexity of the biology of retinal degeneration at late stage disease. To focus our efforts at identifying pathogenic mechanisms that influence retinal degeneration in rcd1 and xlpra2, and likely in other retinal degenerative diseases, only these previously described pathways were further validated at the transcript and protein levels in this study.

**Table 1:** List of canonical pathways identified by IPA that are common between the two diseases and described in this study.

Ingenuity Canonical Pathways	−log(p-value)	
	Rcd1	Xlpra2
Complement System	5.03 ↑	5.82 ↑
Inflammasome pathway	1.26 ↑	1.75 ↑
JAK/Stat Signaling	2.18 ↑	1.68 ↑
NF-κB Signaling	5.29 ↑	5.36 ↑
Prolactin Signaling	2.53 ↑	2.91 ↑
STAT3 Pathway	4.72 ↑	2.54 ↑
TNFR1 Signaling	1.56 ↑	0.795 ↑
TNFR2 Signaling	1.46 ↑	1.72 ↑
Toll-like Receptor Signaling	4.61 ↑	4.57 ↑

Pathways are listed alphabetically; all pathways in this list were predicted to be activated (↑) based on z-score obtained from IPA. The p-value threshold was set at 0.05 (−log(p-value) equivalent 1.3). A complete list of canonical pathways that were selected for IPA canonical pathway analysis for the two mutants is listed in Supplementary Table 3. A more comprehensive analysis with no exclusions is provided in Supplementary Table 4.

### QRT-PCR validation of genes involved in biological pathways identified in late stages of rcd1 and xlpra2

**Figure 3:**
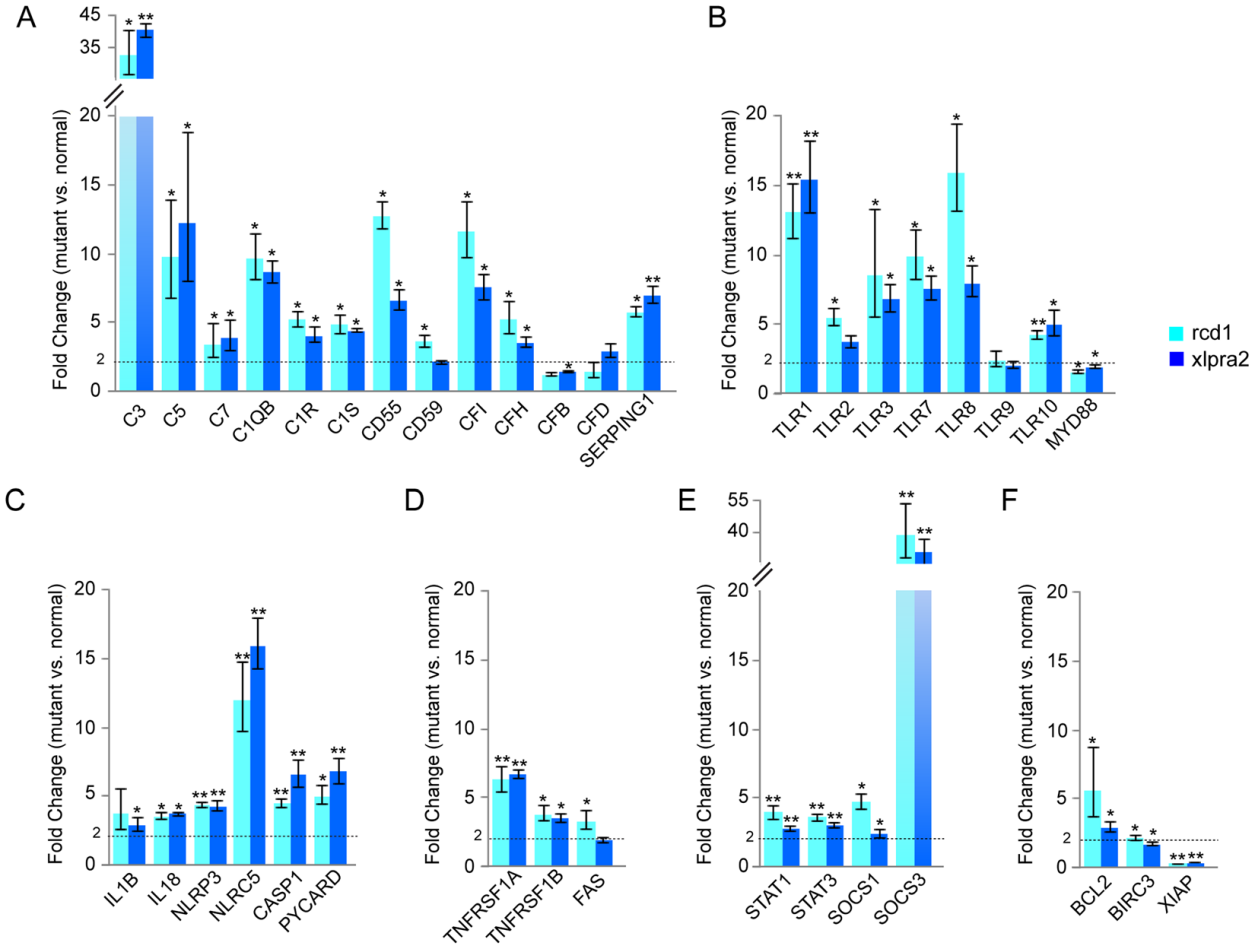
Validation of RNA-seq data by quantitative Real-Time PCR (qRT-PCR) in normal and mutant retinas. Results from qRT-PCR performed on a few select genes from: (**A**) Complement pathway; (**B**) Toll-like receptor family; (**C**) Inflammasome; (**D**) TNF superfamily; (**E**) STAT signaling; and (**F**) Pro-survival genes. The error bars represent the range of the fold change values. Significance is indicated by * for p-value ≤ 0.05 and ** for p-value ≤ 0.001. Dashed line indicates the 2-fold threshold for differentially expressed genes.

To validate the RNA-seq results, quantitative real-time PCR (qRT-PCR) was performed on cDNA from the same retinal tissues to test for expression of a select number of genes representing these functional groups (Fig. 3A–F). Transcripts of several complement components involved in the classical pathway (C1QB, C1R, C1S), common to both the classical and alternative pathways (C3, C5, C7), and inhibitors and regulators of both pathways (CD55, CD59, CFI and CFH) were upregulated in advanced stages of rcd1 and xlpra2 retinal degeneration. In addition, we analyzed the expression of two alternative complement pathway components, complement factor B (CFB) and complement factor D (CFD) that were not identified as differentially regulated by RNA-seq. Expression of CFB mRNA was significantly, albeit only slightly elevated (<2-fold) in xlpra2. The expression of CFD mRNA in the two mutants was not found to be significantly different from that of normal retina (Fig. 3A).

The expression of a number of TLR mRNAs such as TLR1, TLR2, TLR3, TLR7, TLR8, TLR9 and TLR10, as well as adaptor protein myeloid differentiation primary response 88 (Myd88) was found to be increased in both rcd1 and xlpra2 retinas by RNA-seq and validated by qRT-PCR (Fig. 3B). Upregulation of inflammasome components NLRP3 and caspase-1, and pro-inflammatory cytokine IL-18 identified by RNA-seq analysis was also validated by qRT-PCR. Interleukin-1 beta (IL1B) and PYD-and-CARD-Domain-Containing (PYCARD) mRNA, two components of the inflammasome response that were not identified by RNA-seq as being differentially regulated, were found to be upregulated by qRT-PCR in the two diseases (Fig. 3C), although upregulation of IL1B was not significant in rcd1.

The gene expression values for all genes tested in the TNF pathway (TNFRSF1A, TNFRSF1B and FAS) and STAT-pathway (STAT1, STAT3, SOCS1, SOCS3) were found to be in agreement with that obtained by RNA-seq (Fig. 3D and E). With the exception of XIAP, which was found to not be downregulated by qRT-PCR analysis, the gene expression values for other anti-apoptotic genes (BCL2 and BIRC3) also agreed with that obtained by RNA-seq (Fig. 3F). Thus, results from qRT-PCR were consistent with RNA-seq expression results and validated further exploration of these functional pathways.

### Changes in expression of proteins of the innate immune system in degenerated retina

**Figure 4:**
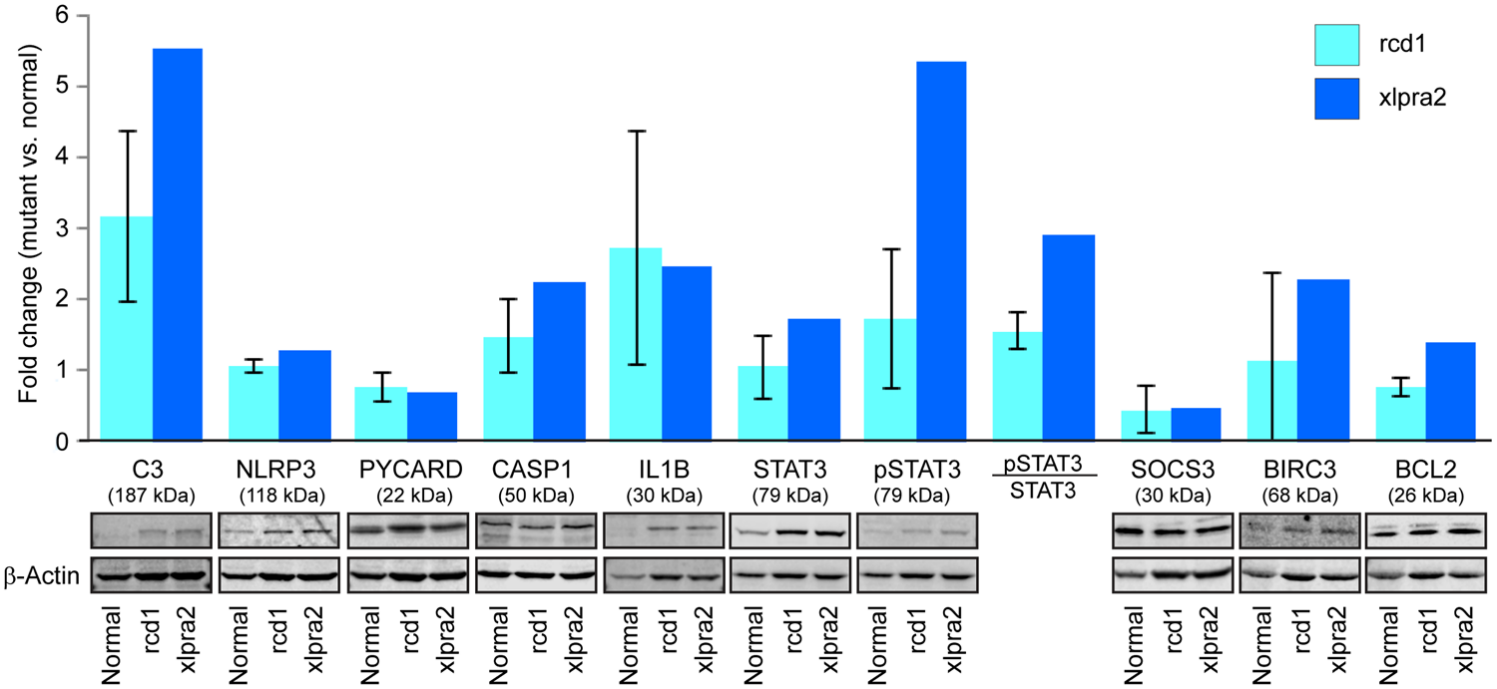
Quantitation of protein levels in the mutant canine retinas in late stages of RP. Representative immunoblots and quantitation of protein levels in mutant (rcd1, n = 3; xlpra2, n = 2) retinas compared to normal retina (n = 3). Error bars represent standard deviation.

To investigate whether the transcriptomic changes translated to similar trends at the protein level, quantitative expression of some of the key pathway proteins was evaluated in the neuroretinal extracts from rcd1 and xlpra2 retinas. Western blot analysis was performed on retinas from a limited number of dogs (3 normal, 3 rcd1 and 2 xlpra2) at ages similar to that of retinas used for transcriptomic profiling. While the results showed a consistent trend in agreement with the transcriptomic data, statistical significance could not be established for any of the proteins tested due to small sample size (Fig. 4). The levels of complement component C3 in both diseases were increased when compared to normal. The mean levels of IL1β protein were also higher in the two disease states, although some individual variation was observed. For CASP1, NLRP3, BCL2 and BIRC3, the increase in protein levels were less pronounced but still reflected the trend observed by qRT-PCR. PYCARD did not show significant increase in the diseased state, while SOCS3 levels were decreased, in contrast with that observed for RNA levels. Total STAT3 was moderately increased in xlpra2 along with an increase in STAT3 phosphorylation.

**Figure 5:**
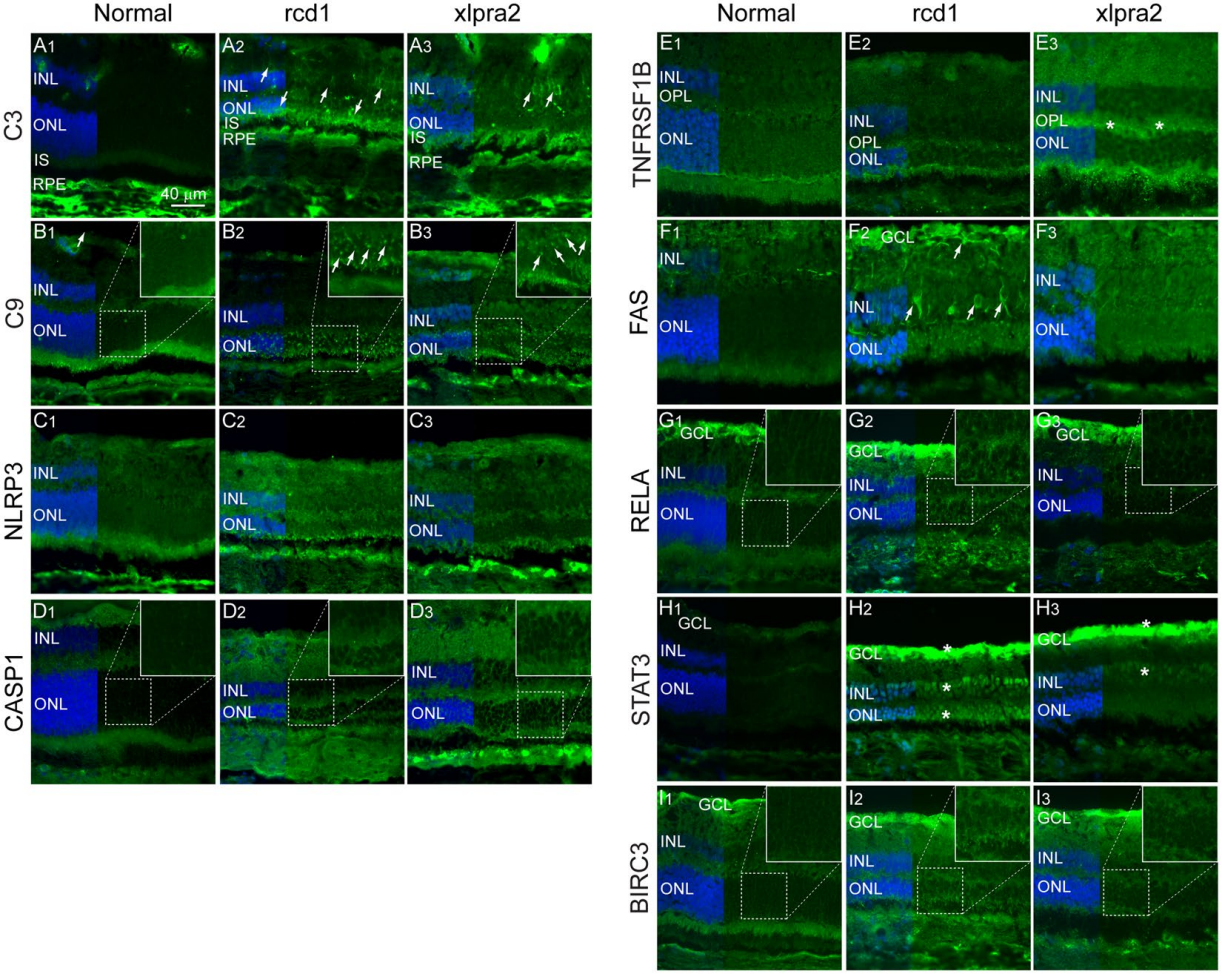
Immunohistochemical localization of select pathway proteins in normal and mutant retina. (**A1–3**) Complement component C3; (**B1–3)** Complement component and component of the membrane attack complex (MAC) C9; (**C1–3)** Inflammasome component NLRP3; (**D1–3)** Apoptosis executor and IL1β activator protein Caspase-1 (CASP1); (**E1–3)** TNF superfamily receptor TNFRSF1B; (**F1–3)** Cell surface death receptor FAS; (**G1–3)** Transcription factor of NFκB family, RELA (p65); (**H1–3)** Transcription activator protein of STAT family, STAT3; (**I1–3)** Anti-apoptotic protein BIRC3. RPE: Retinal Pigment Epithelium; ONL: Outer Nuclear Layer; OPL: Outer Plexiform Layer; INL: Inner Nuclear Layer; GCL: Ganglion Cell Layer.

To investigate whether these transcriptomic changes affected photoreceptor cells and/or other retinal cell populations, the expression and localization of a subset of proteins involved in innate immune response was examined by immunohistochemistry. Complement pathway component C3 was localized in the inner nuclear layer (INL; the layer containing the second order neurons, e.g. bipolar cells, horizontal cells) and photoreceptor inner segments of both rcd1 and xlpra2 retinas, as well as in the ONL of rcd1 retina (Fig. 5A1–3). Complement protein C9, a major component of the membrane attack complex (MAC), formed distinct puncta in the ONL of both mutant retinas, but not in the normal retina (5B1–3).

Increased staining for inflammasome pathway proteins NLRP3 (Nucleotide-binding Oligomerization domain-like (NOD-like) receptor pyrin domain containing 3) was observed in the ONL and the outer plexiform layer (OPL; the synaptic layer between photoreceptors and neurons in the INL) of the two mutant retinas (Fig. 5C1–3). In addition, expression of caspase-1 (CASP1), the caspase responsible for pro-IL1β and pro-IL18 cleavage and activation during inflammation, was also increased in the ONL and INL of mutant retinas (Fig. 5D1–3). Increased expression of TNF superfamily receptor TNFRSF1B was seen in the OPL in the xlpra2 retina (Fig. 5E1–3). Cell surface death receptor FAS showed increased expression in the rcd1 retina in distinct cell populations in the INL and also in ganglion cell layer (GCL) (Fig. 5F1–3).

The expression of NFκB subunit RELA (p65) was found to be slightly elevated in the ONL and INL of rcd1 retina (Fig. 5G1–3). Expression of STAT3 (signal transducer and activator of transcription 3) was found to be high in all three nuclear layers (ONL, INL and GCL) in the mutant animals, and particularly abundant in the ONL of rcd1 retina (Fig. 5H1–3). Finally, slight increase in the immunostaining of anti-apoptotic protein BIRC3 (baculoviral IAP repeat containing 3) was observed in the ONL of mutant retinas, particularly in rcd1 (Fig. 5I1–3). Due to autofluorescence in the RPE, likely caused by accumulation of lipofuscin, this layer could not be evaluated for protein localization in the two diseases. In summary, we confirmed that the transcriptomic changes were frequently paralleled by similar trends at the protein level and changes in protein expression in various retinal layers. Although the two diseases primarily result in death of photoreceptors, significant alterations in other retinal layers occurs possibly as a response to ongoing photoreceptor cell death. The expression of several protein components of the innate immune system in the degenerating retina confirms that activation of innate immunity may play an important role in photoreceptor death in late stage photoreceptor degeneration.

## Discussion

In this study, we present evidence that a number of innate immune pathways and inflammation processes are activated at late stages of two forms of RP with different causative mutations. Inflammation has been shown to play a critical role in pathogenicity of many ocular and retinal diseases such as AMD, DR, uveitis and proliferative vitreoretinopathy. This study now provides evidence that innate immune responses may play an important role in various forms of RP as well.

In recent years, the role of innate immunity and inflammation in mediating cell death after tissue damage has taken prominence in the field of neurodegeneration. Besides its role in self/non-self-discrimination and defense against pathogens, it is now well established that one of the major roles for innate immunity is to sense tissue damage and activate sterile inflammation responses from immune cells. Non-immune cells also participate in sterile immunity through secretion of cytokines and inflammatory mediators, and activation of innate immune receptors^30–32^. While the purpose of an inflammatory response is to eliminate damaged tissues and promote healing, continuous stressors, such as those arising from tissue damage and cell death in inherited degenerative diseases, may lead to a hyperactive and sustained inflammatory response. This can further contribute to disease pathogenicity rather than leading to its resolution. The complement system^33^, pattern recognition receptors such as certain TLRs^16,34^, and NLRP3^13,35^, as well as pro-inflammatory cytokines such as IL-1β, IL-18, IL-33 and TNF-α^36,37^ are all essential mediators of innate immune response and sterile inflammation.

The involvement of both classical and alternative complement pathways is well-established in many ocular and retinal diseases^38^. A number of studies have established a clear association between AMD and complement pathways^12,39,40^. Similarly, increase in components of both the alternative^41,42^ and the classical pathways^43^ is observed in DR. Evidence for alterations in the complement system is not limited to diseases that affect initially the RPE, but is also found in other retinopathies that first involve photoreceptor cells. A transcriptomic study in the rd10 mouse, a model of RP caused by a spontaneous mutation in *Pde6b* gene, at a stage when rod photoreceptors have completely degenerated also revealed an increase in many classical and alternative complement pathway components such as complement components C1Q, C1R, C3 and C4^44^. Similar to these diseases, transcripts of several complement components were upregulated in advanced stages of rcd1 and xlpra2 retinal degeneration. We also found in these retinas a trend suggesting an increase in the protein levels of the pivotal component C3, the site of convergence of the three complement pathways. Although we were unable to confirm formation of the MAC due to lack of canine reactive anti-MAC antibodies (see Supplementary Table S6), staining with anti-C9 antibody, a major protein component of the MAC, showed accumulation of distinct protein aggregates only in the photoreceptor ONL of the diseased animals. This finding strongly suggests that loss of photoreceptors at late stage disease may be MAC-mediated. Thus, involvement of complement proteins in retinal degeneration in the two models of RP explored here parallels similar findings for other retinal diseases. Although the classical complement pathway appears to be the primary mediator of innate immunity in the mutant eyes, we cannot completely exclude the involvement of alternative complement pathway in the two diseases.

The TLRs are pattern recognition receptors that play an important role in the innate immune system by recognizing conserved microbial motifs called pathogen-associated molecular patterns (PAMPs), as well as many endogenous damage-associated molecular patterns (DAMPs), such as high-mobility group B1, heat shock proteins, extracellular matrix components, beta amyloid, and self RNA and DNA^45–47^. Several members of the TLR family have been implicated in sterile immune responses and in the inflammatory component of several associated diseases, including neurodegeneration^48,49^. In addition to their expression on immune cells, TLR expression has been shown in non-immune cells, such as photoreceptors^50^ and RPE cells^51^. In the eye, TLRs have been implicated in retinal ischemia, glaucoma and DR^16^. The expression of a number of TLR mRNAs was found to be increased in both rcd1 and xlpra2 retinas. However, lack of canine-reactive anti-TLR antibodies precluded immunohistochemical determination of the retinal cell populations expressing these TLRs. TLR engagement leads to activation of transcription factors such as MAPK and NFκB (found to be expressed in the INL and ONL of the rcd1 retina) via the adaptor protein myeloid differentiation primary response 88 (Myd88). Activation of NFκB leads to transcription of inflammatory cytokines, including pro-IL1-β and the inflammasome component NLRP3^52^.

This study also identified an upregulation of inflammasome related components NLRP3, PYCARD (ASC), caspase-1, pro-inflammatory cytokines IL-1β, IL-18, IL-33, and the IL-1 receptor (IL-1R) in rcd1 and xlpra2 retinas. In addition, we confirmed increased expression of NLRP3 and caspase-1 proteins in various layers of the mutant retinas. NLRP3 inflammasome activation is a two-step process^53^; the priming signal involves TLR activation or TNF receptor activation that leads to transcription of NLRP3. A second activating signal leads to oligomerization of NLRP3, its assembly with PYCARD (ASC) and pro-caspase-1 to form a functional inflammasome, auto-catalytic activation of caspase-1 and subsequently, formation of mature secreted forms of pro-inflammatory cytokines IL-1β and IL-18^54,55^. Similar to our findings, these inflammation-related molecules are upregulated in other retinal degenerative diseases. In a recent publication, Appelbaum *et al*.^56^ have shown that expression of inflammasome components occurs even earlier in rcd1 and xlpra2, around the peak of cell death^10^ observed in the two diseases. The NLRP3 inflammasome has also been recently implicated in photoreceptor cell death in the Rhodopsin P23H mouse model of RP^57^ and in the *Pde6b* mutant rd1 mouse model^15^. In addition, NLRP3 inflammasome is implicated in disease progression in AMD^58–62^ and DR^63–65^.

It is well established that the TNF pathway is a key mediator of inflammation in many ocular diseases including diabetic retinopathy, uveitis, proliferative vitreoretinopathy, macular edema and wet AMD^17,66^. We previously showed that in the early stages of rcd1 and xlpra2 disease, TNF-superfamily members are upregulated and may thus contribute to the disease pathogenicity^10^. In the current study, continued upregulation of several of the TNF-superfamily genes, including the TNF receptors 1 A and 1B, and cell death receptor FAS, was observed at late stages of the two diseases; however, unlike at the earlier stages, levels of TNF-α were not significantly elevated in the late stage retina. This suggests that targeting TNF-α may be a valid therapeutic strategy but only earlier in the course of photoreceptor degeneration.

An initial acute burst of photoreceptor cell death followed by a protracted death of remaining photoreceptors that extends over several weeks or months has been reported in several rodent and canine models of RP^9,10,29^. While the deleterious effects of the mutations maybe directly responsible for the early loss of rods^67,68^, the second phase of slow cell loss may be the result of an interplay between photoreceptor survival pathways and chronic inflammatory processes. Increased expression of anti-apoptotic protein BIRC3 in the ONL and increase in BCL2 mRNA was observed in both rcd1 and xlpra2 retinas. On the other hand, neurotrophic factors such as CNTF, GDNF, BDNF, NGF and bFGF that are known to improve photoreceptor survival^69^, were not significantly increased. Noteworthy, however, was the very significant upregulation of the prolactin hormone mRNA, which has been shown to play a protective role in retinal degeneration^70–72^ (Supplementary Table S2). Our transcriptomic data also revealed an increase in a number of non-apoptotic pathway genes that have been implicated in retinal degeneration, such as a number of poly-ADP-ribose-polymerases (PARP9, PARP14, PARP15) and histone deacetylases (HDAC7, HDAC10, HDAC11).

Availability of a low number of animals at advanced stages of disease, particularly of xlpra2 dogs, was a limitation of this study. There was, however, some concordant trends in mRNA and protein level changes, in particular for components of the complement and inflammasome pathways. Even though the two diseases result primarily in a loss of photoreceptors, the inflammatory responses appear to involve other retinal cell populations as expression of several of the innate immune proteins occurred in the inner retina. The role of microglia and inflammatory cells such as macrophages has been described for several retinal degenerative models^44,73–78^. The contribution of neuronal cells as well as microglia to inflammation in late stages of RP will be the subject of future investigations. Although we expected to see some of the inflammation pathways to be active in RPE cells as well, increased autofluorescence presumably due to lipofuscin accumulation^79^ in the RPE cells in the late stages of the two diseases precluded further immunohistochemical evaluation of this layer.

In summary, our results indicate that more than one innate immune response pathway is activated in both diseases at advanced stages of degeneration. This work, complements two other studies^10,56^ that assessed by qRT-PCR in early stages of rcd1 and xlpra2 diseases the expression of a limited number of pre-selected genes involved in inflammatory pathways. Taken together these results suggest that the transcriptomic profile of a retina undergoing photoreceptor loss evolves with the stage of degeneration. For example, while the inflammasome pathway appears to be activated throughout early to late disease^56^, increased expression of TNF-α was only found shortly after disease onset^10^. In addition, as this current study utilized RNA-seq technology, it provided a considerably more comprehensive analysis of the biological pathways involved in late stage retinal degeneration. As a result, we were able to identify upregulated genes (e.g. components of the complement pathway) not previously reported in these models of RP. Presence of multiple inflammation pathways in the two diseases is expected as these various components of the inflammatory pathways do not work in isolation but stimulate and augment each other’s activation and function. Parallels have been shown in AMD where C1q, a complement protein known to be a component of AMD drussen, is known to activate NLRP3 function and stimulate IL-1β and IL-18 secretion^80^. Formation of MAC at sub-lethal levels also activates NLRP3 inflammasome^81^.

In conclusion, this is the first study comparing the retinal transcriptome of two non-allelic animal models of late stage RP. The results of this work, conducted in two naturally-occurring canine models, suggest that the involvement of the innate immune system may be a common response in late stage RP and, while localized to other retinal layers than the RPE, shows striking similarities with the profile of AMD and DR. A number of pharmacological therapeutics currently on the market or in development for these common retinopathies target inflammation molecules such as complement pathway proteins^12^ and IL-1β^82^ and thus opens the possibility for also testing similar strategies for the treatment of late stage RP.

## Methods

### Animals

Retinas from five female normal dogs (age: 24–26 weeks), six female rcd1 affected dogs (age: 22 weeks) and five female xlpra2 affected dogs (age: 41 weeks) were used (Supplementary Table S1) for this study. Mutant animals were selected at an age where the ONL thickness in the retina had decreased to less than 50% of that of normal dogs. All dogs were housed under identical conditions (diet, ambient illumination with cyclic 12 hrs ON-12 hrs OFF light) at the Retinal Disease Studies (RDS) facility at the University of Pennsylvania. The studies strictly adhered to the ARVO Statement for the Use of Animals in Ophthalmic and Vision Research and were approved by the Institutional Animal Care and Use Committee (IACUC) of the University of Pennsylvania. Dogs were euthanized with an intravenous injection of sodium pentobarbital. After enucleation, the neuroretina was removed and stored at −80 °C until further experiments, or the posterior eyecup dissected into four quadrants and embedded in Optimal Cutting Temperature (OCT) medium without prior fixation.

### RNA isolation

Neuroretinas from 3 normal, 3 rcd1, and 3 xlpra2 dogs were homogenized in TRIzol (Invitrogen, Thermo Fisher Scientific) using the BeadBug microtube homogenizer (Benchmark Scientific) and total RNA was extracted using the standard Trizol extraction protocol. RNA was treated with Turbo DNA-free kit (Thermo Fisher Scientific) to remove any genomic DNA contamination. RNA was quantified using the Nanodrop-1000 (ND-1000) spectrophotometer (Nanodrop Technologies, Thermo Scientific) and the quality of RNA was assessed with an Agilent 2100 Bioanalyzer (Agilent Technologies). All RNA samples had a RIN number >9 and were used for preparation of a cDNA library for RNA-seq.

### cDNA library preparation, RNA-seq and analysis

A cDNA library from the 9 samples was prepared using Illumina Truseq mRNA library prep kit v2 and used for 100 bp single read sequencing on an Illumina Hiseq 2000 device. The resulting sequence data were recorded in FASTQ format (GEO accession GSE97638). Only high-quality reads were retained and any reads with a poor quality run of ≥5 were eliminated. Reads that aligned to a generic set of sequences containing ribosomal and known repeat sequences in humans were also removed before further analysis of the sequencing data. RNA-seq Unified Mapper (RUM)^83^ was used for aligning reads to known transcripts included in RefSeq, UCSC known genes and ENSEMBL transcripts, and to the dog genome. Unique alignments to RefSeq were extracted from the RUM output. Statistical and graphical analysis of RNA-seq data was carried out using R/Bioconductor. Differential gene expression analysis was performed using EdgeR package from R/Bioconductor^84^. The p-values were corrected for multiple testing using the Benjamini and Hochberg mode of the R function *p.adjust* to compute the false discovery rate. Heatmaps were generated using *heatmaps.2* function of *gplots* package in R/Bioconductor. Differentially expressed genes (DEGs) with a p ≤ 0.05 and at least a 2-fold (±) difference in expression compared to normal were considered for further analysis. Annotation and pathway analysis on differentially expressed genes was performed using Ingenuity Pathway Analysis (Ingenuity® Systems, Spring Release 2017, www.ingenuity.com). Pathways with a −log(p-value) score of minimum 1.3 (Fisher’s exact test) and that were relevant to retinal degeneration were selected for further characterization.

### qRT-PCR

Expression of select genes in each of the 9 RNA samples (3 per group) was further examined using qRT-PCR. Total RNA was reverse transcribed into cDNA using the High Capacity RNA to cDNA kit (Applied Biosystems, Thermo Fisher Scientific). Gene-specific PCR primer pairs were designed using the free PrimerQuest Tool from Integrated DNA Technologies. Primers used for qRT-PCR have been listed in Supplementary Table S5. qRT-PCR was performed either on a 7500 Fast Real-Time PCR system (96-well format) or ViiA 7 Real-Time PCR System (348-well format) (Applied Biosystems, Thermo Fisher Scientific). Each 20 ul reaction contained 5 ng of cDNA, 10 ul of SYBR Green PCR Mastermix (Life Technologies, Thermo Fisher Scientific) and 250 nM each forward and reverse primers. Each sample was analyzed in triplicates. Comparative deltaCt method was used for relative comparison of gene expression levels using GAPDH as an endogenous control for normalization. Fold change was calculated as 2^−(ΔΔCt)^ and statistical significance was calculated by an unpaired homoscedastic t-test using a two-tailed distribution.

### Retinal morphology and morphometry

H&E stained retinal cryosections (12 µm-thick) extending from the optic nerve head (ONH) to the *ora serrata* along the 4 meridians (superior, inferior, nasal and temporal) were examined under bright field microscopy (Axioplan, Carl Zeiss Meditec GmbH, Oberkochen, Germany) with a 40X objective. Number of ONL nuclei were counted every 1,000 microns and the ONL thickness was plotted on biaxial (superior/inferior and nasal/temporal) spidergraphs.

### Western blot analysis

Neuroretinal extracts were prepared from 3 normal, 3 rcd1 and 2 xlpra2 retinas in a buffer containing 0.23 M sucrose, 2 mM EDTA, 5 mM Tris HCl, pH 7.4, 1% Tx100 and a protease and phosphatase inhibitor cocktail (Halt, Thermo Scientific, Waltham, MA). The retinas were first homogenized by vortexing with 1.5 mm zirconium beads (Benchmark Scientific Inc., Sayreville, NJ) and then sonicated. The samples were then centrifuged and total protein concentration in the supernatant measured by BCA assay. 50 µgs of total protein from each sample was resolved on an 8–16% Tris Glycine gel (Invitrogen, Carlsbad, CA), transferred to a nitrocellulose membrane (iBLOT, Invitrogen) and immunoblotted using antibodies listed in Supplementary Table 6. After incubation with infrared dye-tagged secondary antibodies, protein bands were visualized on a digital imaging system (Odyssey Fc, Licor, Lincoln, NE). Quantification was performed using the Licor Image Studio v4.0 software using β-actin bands for normalization. Statistical significance was calculated for rcd1 samples only by unpaired homoscedastic t-test using a two-tailed distribution.

### Immunohistochemistry

Expression and localization of select proteins was analyzed by immunohistochemistry using a panel of antibodies (listed in Supplementary Table S6) on 12 µm-thick retinal sections from the superior part of retina. The sections were incubated with primary antibodies at 4 °C overnight after a blocking step with buffer containing 5% BSA and 4.5% fish gelatin in PBS at room temperature. Antigen antibody complexes were visualized with Alexa-fluor labeled secondary antibodies (Invitrogen, Thermo Fisher Scientific). DAPI stain was used to label the nuclei. After mounting in Gelvatol Mounting medium (containing polyvinyl alcohol and glycerol), slides were examined with an epifluorescence microscope (Axioplan, Carl Zeiss Meditec), images were digitally captured using Spot 4.0 camera and prepared for display using Adobe Photoshop and Illustrator programs.

## Electronic supplementary material

Supplementary Information

Supplementary Table 2

## Data Availability

Transcriptomic data (RNA-seq) generated for this study are available in the GEO repository (accession number GSE97638). Ingenuity pathway analysis data are provided either as Supplementary information file or available from the corresponding author on reasonable request.
