# Author Correction: Involvement of Innate Immune System in Late Stages of Inherited Photoreceptor Degeneration

**DOI:** 10.1038/s41598-018-35520-2

**Published:** 2018-11-14

**Authors:** Raghavi Sudharsan, Daniel P. Beiting, Gustavo D. Aguirre, William A. Beltran

**Affiliations:** 1 Division of Experimental Retinal Therapies, Department of Clinical Sciences and Advanced Medicine, School of Veterinary Medicine, University of Pennsylvania, Philadelphia, PA 19104 USA; 2 Department of Pathobiology, School of Veterinary Medicine, University of Pennsylvania, Philadelphia, PA 19104 USA

Correction to: *Scientific Reports* 10.1038/s41598-017-18236-7, published online 20 December 2017

The original version of this Article contained an error in the Abstract.

“Over 200 mutations in 60 different genes have been shown to cause RP.”

now reads:

“Many mutations in 60 different genes have been shown to cause RP.”

