# Supplementary material for: Involvement of Innate Immune System in Late Stages of Inherited Photoreceptor Degeneration
Source: Sci Rep. 2017 Dec 20;7:17897. doi: 10.1038/s41598-017-18236-7 (PMC5738376; doi:10.1038/s41598-017-18236-7)

Supplementary Information

Involvement of Innate Immune System in Late Stages of Inherited Photoreceptor Degeneration

Raghavi Sudharsan, Daniel P. Beiting, Gustavo D. Aguirre, William A. Beltran

Supplementary Table 1: Summary of dogs used for this study

**Dog ID**	**Phenotype**	**Age (weeks)**	**Sex**	**Experiment**
CEACMI	Normal	24	F	OS: RNA extraction, RNA-seq, qPCR
CEACID	Normal	24	F	OS: RNA extraction, RNA-seq, qPCR
CEACIV	Normal	24	F	OD: Western blot analysis<br>OS: RNA extraction, RNA-seq, qPCR
CGBCHK	Normal	26	F	OD: OCT embedding without fixation, histology, immunohistochemistry<br>OS: Western blot analysis.
CGBCHL	Normal	26	F	OD: Western blot analysis
2149	rcd1 affected	22	F	OD: OCT embedding without fixation, histology, immunohistochemistry<br>OS: RNA extraction, RNA-seq, qPCR
2150	rcd1 affected	22	F	OD: OCT embedding without fixation, histology, immunohistochemistry<br>OS: RNA extraction, RNA-seq, qPCR
2151	rcd1 affected	22	F	OD: OCT embedding without fixation, histology, immunohistochemistry<br>OS: RNA extraction, RNA-seq, qPCR
2225	rcd1 affected	22	M	OS: Western blot analysis
2226	rcd1 affected	22	F	OS: Western blot analysis
2227	rcd1 affected	22	F	OS: Western blot analysis
Z478	xlpra2 affected	41	F	OD: OCT embedding without fixation, histology, immunohistochemistry<br>OS: RNA extraction, RNA-seq, qPCR
Z479	xlpra2 affected	41	F	OD: OCT embedding without fixation, histology, immunohistochemistry<br>OS: RNA extraction, RNA-seq, qPCR
Z480	xlpra2 affected	41	F	OD: OCT embedding without fixation, histology, immunohistochemistry<br>OS: RNA extraction, RNA-seq, qPCR
Z598	xlpra2 affected	41	F	OS: Western blot analysis
Z599	xlpra2 affected	41	F	OS: Western blot analysis

Supplementary Table 3: List of canonical pathways with identified by IPA with -log(p-value) ≥ 1.3*. Pathways are listed in the order of their –log(p-value); the arrow next to the –log(p-value) indicates whether the pathway was predicted to be activated (↑) or inhibited (↓) by IPA (z-score). If no arrow is indicated, it means IPA did not make any prediction about the activation state of the pathway. Pathways common between rcd1 and xlpra2 inhibited are highlighted in blue. Pathways that are further described in this study and common between the two diseases are highlighted in green.

* Inflammasome pathway in rcd1 falls a little under the p-value threshold.

**Rcd1**		**Xlpra2**	
**Ingenuity Canonical Pathways**	**-log(p-value)**	**Ingenuity Canonical Pathways**	**-log(p-value)**
Axonal Guidance Signaling	19.0	Phototransduction Pathway	10.2
RhoGDI Signaling	9.82 ↓	TREM1 Signaling	9.25 ↑
Signaling by Rho Family GTPases	8.75 ↑	Coagulation System	7.99 ↓
Phototransduction Pathway	8.46	Axonal Guidance Signaling	7.63
Endothelin-1 Signaling	7.53 ↑	Phospholipase C Signaling	7.52 ↑
IL-8 Signaling	7.52 ↑	Th1 and Th2 Activation Pathway	7.44
Phospholipase C Signaling	7.33 ↑	LXR/RXR Activation	7.18 ↓
Leukocyte Extravasation Signaling	7.28 ↑	Communication between Innate and Adaptive Immune Cells	6.94
Th1 and Th2 Activation Pathway	6.86	Crosstalk between Dendritic Cells and Natural Killer Cells	6.94
TREM1 Signaling	6.68 ↑	T Helper Cell Differentiation	6.57
cAMP-mediated signaling	6.68 ↑	Acute Phase Response Signaling	6.46 ↑
G-Protein Coupled Receptor Signaling	6.57	Leukocyte Extravasation Signaling	6.44 ↑
Ephrin Receptor Signaling	6.54 ↑	Dendritic Cell Maturation	6.18 ↑
LXR/RXR Activation	6.51 ↓	Interferon Signaling	5.98 ↑
Ephrin B Signaling	6.37 ↑	Complement System	5.82 ↑
Gαi Signaling	6.14 ↑	Th2 Pathway	5.67 ↑
Inhibition of Matrix Metalloproteases	6.09	Tec Kinase Signaling	5.45 ↑

Tec Kinase Signaling	6.05 ↑	Phagosome Formation	5.44
Thrombin Signaling	5.88 ↑	Role of JAK1 and JAK3 in γc Cytokine Signaling	5.44
CXCR4 Signaling	5.64 ↑	Endothelin-1 Signaling	5.44 ↑
Acute Phase Response Signaling	5.34 ↑	LPS/IL-1 Mediated Inhibition of RXR Function	5.40 ↑
NF-κB Signaling	5.29 ↑	NF-κB Signaling	5.36 ↑
Th1 Pathway	5.22 ↑	Th1 Pathway	5.12 ↑
Relaxin Signaling	5.07 ↑	Role of NFAT in Regulation of the Immune Response	5.10 ↑
Complement System	5.03 ↑	ILK Signaling	4.99 ↑
Crosstalk between Dendritic Cells and Natural Killer Cells	4.99	Caveolar-mediated Endocytosis Signaling	4.82
Dendritic Cell Maturation	4.97 ↑	Toll-like Receptor Signaling	4.57 ↑
Role of NFAT in Regulation of the Immune Response	4.95 ↑	Intrinsic Prothrombin Activation Pathway	4.52 ↑
Th2 Pathway	4.84 ↑	RhoGDI Signaling	4.42 ↓
Synaptic Long Term Depression	4.75 ↑	Signaling by Rho Family GTPases	4.34 ↑
T Helper Cell Differentiation	4.72	Death Receptor Signaling	4.34 ↑
STAT3 Pathway	4.72 ↑	HMGB1 Signaling	4.29 ↑
LPS/IL-1 Mediated Inhibition of RXR Function	4.67 ↑	IL-8 Signaling	4.14 ↑
Toll-like Receptor Signaling	4.61 ↑	Synaptic Long Term Depression	4.05 ↑
Paxillin Signaling	4.55 ↑	Eicosanoid Signaling	3.96 ↑
Communication between Innate and Adaptive Immune Cells	4.51	CD28 Signaling in T Helper Cells	3.95 ↑
Gαq Signaling	4.50 ↑	PKCθ Signaling in T Lymphocytes	3.90 ↑
Wnt/Ca+ pathway	4.49 ↑	Oncostatin M Signaling	3.86 ↑
Protein Kinase A Signaling	4.48 ↑	T Cell Receptor Signaling	3.78
Gαs Signaling	4.47 ↑	Protein Kinase A Signaling	3.76 ↑
ILK Signaling	4.26 ↑	IL-6 Signaling	3.72 ↑
Phagosome Formation	4.24	Epithelial Adherens Junction Signaling	3.64
Caveolar-mediated Endocytosis Signaling	3.94	Production of Nitric Oxide and Reactive Oxygen Species in Macrophages	3.56 ↑
Role of JAK1 and JAK3 in γc Cytokine Signaling	3.94	Thrombin Signaling	3.54 ↑
P2Y Purigenic Receptor Signaling Pathway	3.89 ↑	iCOS-iCOSL Signaling in T Helper Cells	3.54 ↑

Interferon Signaling	3.86 ↑	Calcium Signaling	3.43 ↑
HMGB1 Signaling	3.82 ↑	Relaxin Signaling	3.37 ↑
TGF-β Signaling	3.81 ↑	IL-10 Signaling	3.34
G Beta Gamma Signaling	3.72	TGF-β Signaling	3.15 ↑
FAK Signaling	3.64	Chemokine Signaling	3.14 ↑
Integrin Signaling	3.63 ↑	Extrinsic Prothrombin Activation Pathway	3.13
IL-6 Signaling	3.51 ↑	Clathrin-mediated Endocytosis Signaling	3.07
Macropinocytosis Signaling	3.47 ↑	B Cell Development	3.05
Fcγ Receptor-mediated Phagocytosis in Macrophages and Monocytes	3.32 ↑	Apoptosis Signaling	3.04 ↑
IL-10 Signaling	3.31	Regulation of Cellular Mechanics by Calpain Protease	3.03 ↑
CCR5 Signaling in Macrophages	3.22	3-phosphoinositide Biosynthesis	3.00
Actin Cytoskeleton Signaling	3.20 ↑	Integrin Signaling	2.98 ↑
fMLP Signaling in Neutrophils	3.20 ↑	Calcium-induced T Lymphocyte Apoptosis	2.95 ↑
Antiproliferative Role of Somatostatin Receptor 2	3.13 ↑	p38 MAPK Signaling	2.94 ↑
Calcium Signaling	3.12 ↑	Prolactin Signaling	2.91 ↑
Sphingosine-1-phosphate Signaling	3.08 ↑	Gαs Signaling	2.90 ↑
Aryl Hydrocarbon Receptor Signaling	3.05	cAMP-mediated signaling	2.86 ↑
Chemokine Signaling	3.04 ↑	Fcγ Receptor-mediated Phagocytosis in Macrophages and Monocytes	2.83 ↑
Wnt/β-catenin Signaling	3.00 ↓	IL-9 Signaling	2.80 ↑
PTEN Signaling	2.99 ↓	CCR5 Signaling in Macrophages	2.77
Gα12/13 Signaling	2.98 ↑	Paxillin Signaling	2.72 ↑
Regulation of Cellular Mechanics by Calpain Protease	2.97 ↑	Wnt/β-catenin Signaling	2.71 ↓
Phospholipases	2.96	Gαq Signaling	2.69 ↑
Serotonin Receptor Signaling	2.95	Cytotoxic T Lymphocyte-mediated Apoptosis of Target Cells	2.68
Gap Junction Signaling	2.95	Sphingosine-1-phosphate Signaling	2.68 ↑

Renin-Angiotensin Signaling	2.93 ↑	FXR/RXR Activation	2.56
FXR/RXR Activation	2.90	CTLA4 Signaling in Cytotoxic T Lymphocytes	2.54
PKCθ Signaling in T Lymphocytes	2.87 ↑	Ephrin Receptor Signaling	2.54 ↑
IL-9 Signaling	2.73 ↑	STAT3 Pathway	2.54 ↑
Epithelial Adherens Junction Signaling	2.72	Wnt/Ca+ pathway	2.51 ↑
L-1 Signaling	2.71 ↑	Glucocorticoid Receptor Signaling	2.49
Production of Nitric Oxide and Reactive Oxygen Species in Macrophages	2.68 ↑	Actin Cytoskeleton Signaling	2.42 ↑
p70S6K Signaling	2.62 ↑	PPAR Signaling	2.41 ↓
PPAR Signaling	2.58 ↓	Nur77 Signaling in T Lymphocytes	2.38
Eicosanoid Signaling	2.55 ↑	Gap Junction Signaling	2.35
Prolactin Signaling	2.53 ↑	G-Protein Coupled Receptor Signaling	2.35
iCOS-iCOSL Signaling in T Helper Cells	2.50 ↑	Phospholipases	2.22
RhoA Signaling	2.50 ↑	CXCR4 Signaling	2.20 ↑
Apoptosis Signaling	2.49 ↑	Inhibition of Matrix Metalloproteases	2.12
ERK5 Signaling	2.46 ↑	IL-2 Signaling	2.11 ↑
Semaphorin Signaling in Neurons	2.44	Ephrin B Signaling	2.10 ↑
PAK Signaling	2.42 ↑	Renin-Angiotensin Signaling	2.07 ↑
Oncostatin M Signaling	2.40 ↑	Reelin Signaling in Neurons	2.06
Netrin Signaling	2.37	Aryl Hydrocarbon Receptor Signaling	2.05
Regulation of Actin-based Motility by Rho	2.36 ↑	IL-3 Signaling	2.05 ↑
CD28 Signaling in T Helper Cells	2.32 ↑	Role of JAK family kinases in IL-6-type Cytokine Signaling	2.04
Reelin Signaling in Neurons	2.30	P2Y Purigenic Receptor Signaling Pathway	2.00 ↑
Death Receptor Signaling	2.30 ↑	IL-15 Signaling	1.96
B Cell Development	2.29	Role of JAK2 in Hormone-like Cytokine Signaling	1.92
T Cell Receptor Signaling	2.28	VDR/RXR Activation	1.87 ↑
BMP signaling pathway	2.28 ↑	ErbB2-ErbB3 Signaling	1.87 ↑
IL-3 Signaling	2.18 ↑	Tight Junction Signaling	1.85

JAK/Stat Signaling	2.18 ↑	Regulation of IL-2 Expression in Activated and Anergic T Lymphocytes	1.83
CREB Signaling in Neurons	2.08 ↑	FAK Signaling	1.79
Melatonin Degradation II	2.05	FcγRIIB Signaling in B Lymphocytes	1.78
Clathrin-mediated Endocytosis Signaling	2.04	Inflammasome pathway	1.75 ↑
FcγRIIB Signaling in B Lymphocytes	2.03 ↑	Macropinocytosis Signaling	1.75 ↑
GADD45 Signaling	2.02	ERK5 Signaling	1.73 ↑
Notch Signaling	2.00 ↑	TNFR2 Signaling	1.72 ↑
ERK/MAPK Signaling	1.97 ↑	JAK/Stat Signaling	1.68 ↑
ErbB Signaling	1.97	Thrombopoietin Signaling	1.64 ↑
Ceramide Signaling	1.93 ↑	Actin Nucleation by ARP-WASP Complex	1.64 ↑
Intrinsic Prothrombin Activation Pathway	1.93 ↑	Netrin Signaling	1.61
CDK5 Signaling	1.91 ↑	IL-12 Signaling and Production in Macrophages	1.58
Ephrin A Signaling	1.91	ERK/MAPK Signaling	1.58 ↑
GNRH Signaling	1.88 ↑	PPARα/RXRα Activation	1.57 ↓
Role of JAK family kinases in IL-6-type Cytokine Signaling	1.84	Sumoylation Pathway	1.56 ↑
IL-4 Signaling	1.84	G Beta Gamma Signaling	1.50 ↑
Retinol Biosynthesis	1.83	Fc Epsilon RI Signaling	1.47 ↑
Coagulation System	1.82 ↓	PTEN Signaling	1.47 ↓
VDR/RXR Activation	1.82	Calcium Transport I	1.47
B Cell Receptor Signaling	1.82 ↑	OX40 Signaling Pathway	1.46
Actin Nucleation by ARP-WASP Complex	1.81 ↑	IL-4 Signaling	1.46
ErbB2-ErbB3 Signaling	1.69 ↑	Gα12/13 Signaling	1.46 ↑
IL-12 Signaling and Production in Macrophages	1.68	Gαi Signaling	1.45 ↑
G Protein Signaling Mediated by Tubby	1.64	PDGF Signaling	1.43 ↑
Telomerase Signaling	1.63 ↑	fMLP Signaling in Neutrophils	1.42 ↑
IL-15 Signaling	1.62	B Cell Receptor Signaling	1.41 ↑
Thrombopoietin Signaling	1.60 ↑	TWEAK Signaling	1.41 ↑
14-3-3-mediated Signaling	1.59 ↑	Regulation of Actin-based Motility by Rho	1.40
April Mediated Signaling	1.57 ↑	IL-1 Signaling	1.40 ↑
Angiopoietin Signaling	1.57 ↑	Serotonin Receptor Signaling	1.40

TNFR1 Signaling	1.56 ↑
Fc Epsilon RI Signaling	1.55 ↑
GABA Receptor Signaling	1.49
Role of JAK2 in Hormone-like Cytokine Signaling	1.48
Erythropoietin Signaling	1.47
TNFR2 Signaling	1.46 ↑
Role of JAK1, JAK2 and TYK2 in Interferon Signaling	1.45 ↑
Calcium Transport I	1.45
PPARα/RXRα Activation	1.43 ↓
TR/RXR Activation	1.42
CTLA4 Signaling in Cytotoxic T Lymphocytes	1.38
α-Adrenergic Signaling	1.38 ↑
IL-2 Signaling	1.35 ↑
Neurotrophin/TRK Signaling	1.33 ↑
Neuregulin Signaling	1.33 ↑
Nur77 Signaling in T Lymphocytes	1.3
Inflammasome pathway*	1.26* ↑

Ceramide Signaling	1.34 ↑
IL-15 Production	1.32

Supplementary Table 4: Complete set of canonical pathways identified by IPA for rcd1 and xlpra2. Pathways common to rcd1 and xlpra2 are highlighted in blue. All pathways, including metabolic and cancer pathways, are included.

rcd1		xlpra2	
**Ingenuity Canonical Pathways**	**-log(p-value)**	**Ingenuity Canonical Pathways**	**-log(p-value)**
Axonal Guidance Signaling	19	Hepatic Fibrosis / Hepatic Stellate Cell Activation	11.5
Hepatic Fibrosis / Hepatic Stellate Cell Activation	18.8	Phototransduction Pathway	10.2
Role of Osteoblasts, Osteoclasts and Chondrocytes in Rheumatoid Arthritis	10	Role of Macrophages, Fibroblasts and Endothelial Cells in Rheumatoid Arthritis	9.66
RhoGDI Signaling	9.82	TREM1 Signaling	9.25
Colorectal Cancer Metastasis Signaling	9.58	Role of Osteoblasts, Osteoclasts and Chondrocytes in Rheumatoid Arthritis	8.96
Role of Macrophages, Fibroblasts and Endothelial Cells in Rheumatoid Arthritis	9.46	Role of Pattern Recognition Receptors in Recognition of Bacteria and Viruses	8.81
Signaling by Rho Family GTPases	8.75	Atherosclerosis Signaling	8.47
Phototransduction Pathway	8.46	Colorectal Cancer Metastasis Signaling	8.06
Molecular Mechanisms of Cancer	8.28	Coagulation System	7.99
Atherosclerosis Signaling	7.85	Granulocyte Adhesion and Diapedesis	7.98
Endothelin-1 Signaling	7.53	Axonal Guidance Signaling	7.63
IL-8 Signaling	7.52	Phospholipase C Signaling	7.52
Phospholipase C Signaling	7.33	Th1 and Th2 Activation Pathway	7.44
Leukocyte Extravasation Signaling	7.28	LXR/RXR Activation	7.18
Granulocyte Adhesion and Diapedesis	7.12	Communication between Innate and Adaptive Immune Cells	6.94
Altered T Cell and B Cell Signaling in Rheumatoid Arthritis	6.96	Crosstalk between Dendritic Cells and Natural Killer Cells	6.94

Th1 and Th2 Activation Pathway	6.86	T Helper Cell Differentiation	6.57
Human Embryonic Stem Cell Pluripotency	6.68	Antigen Presentation Pathway	6.52
TREM1 Signaling	6.68	Acute Phase Response Signaling	6.46
cAMP-mediated signaling	6.68	Leukocyte Extravasation Signaling	6.44
G-Protein Coupled Receptor Signaling	6.57	Agranulocyte Adhesion and Diapedesis	6.23
Ephrin Receptor Signaling	6.54	Altered T Cell and B Cell Signaling in Rheumatoid Arthritis	6.22
LXR/RXR Activation	6.51	Dendritic Cell Maturation	6.18
Ephrin B Signaling	6.37	Interferon Signaling	5.98
Role of Pattern Recognition Receptors in Recognition of Bacteria and Viruses	6.34	Complement System	5.82
Agranulocyte Adhesion and Diapedesis	6.15	Type I Diabetes Mellitus Signaling	5.76
Gαi Signaling	6.14	Th2 Pathway	5.67
Inhibition of Matrix Metalloproteases	6.09	Molecular Mechanisms of Cancer	5.54
Tec Kinase Signaling	6.05	Tec Kinase Signaling	5.45
Thrombin Signaling	5.88	Phagosome Formation	5.44
CXCR4 Signaling	5.64	Role of JAK1 and JAK3 in γc Cytokine Signaling	5.44
Basal Cell Carcinoma Signaling	5.37	Endothelin-1 Signaling	5.44
Acute Phase Response Signaling	5.34	LPS/IL-1 Mediated Inhibition of RXR Function	5.4
Adipogenesis pathway	5.3	NF-κB Signaling	5.36
NF-κB Signaling	5.29	Th1 Pathway	5.12
Th1 Pathway	5.22	Role of NFAT in Regulation of the Immune Response	5.1
Agrin Interactions at Neuromuscular Junction	5.19	ILK Signaling	4.99
Relaxin Signaling	5.07	Caveolar-mediated Endocytosis Signaling	4.82
Regulation of the Epithelial-Mesenchymal Transition Pathway	5.04	Toll-like Receptor Signaling	4.57
Complement System	5.03	Intrinsic Prothrombin Activation Pathway	4.52

Crosstalk between Dendritic Cells and Natural Killer Cells	4.99	Regulation of the Epithelial-Mesenchymal Transition Pathway	4.5
Dendritic Cell Maturation	4.97	RhoGDI Signaling	4.42
Role of NFAT in Regulation of the Immune Response	4.95	Signaling by Rho Family GTPases	4.34
Th2 Pathway	4.84	Death Receptor Signaling	4.34
PCP pathway	4.84	HMGB1 Signaling	4.29
Synaptic Long Term Depression	4.75	Human Embryonic Stem Cell Pluripotency	4.21
T Helper Cell Differentiation	4.72	Basal Cell Carcinoma Signaling	4.15
STAT3 Pathway	4.72	IL-8 Signaling	4.14
LPS/IL-1 Mediated Inhibition of RXR Function	4.67	Superpathway of Inositol Phosphate Compounds	4.13
Toll-like Receptor Signaling	4.61	PI3K Signaling in B Lymphocytes	4.11
Paxillin Signaling	4.55	Synaptic Long Term Depression	4.05
Communication between Innate and Adaptive Immune Cells	4.51	Eicosanoid Signaling	3.96
Gαq Signaling	4.5	CD28 Signaling in T Helper Cells	3.95
Wnt/Ca+ pathway	4.49	PKCθ Signaling in T Lymphocytes	3.9
Protein Kinase A Signaling	4.48	Oncostatin M Signaling	3.86
Gαs Signaling	4.47	T Cell Receptor Signaling	3.78
Cardiac Hypertrophy Signaling	4.26	Graft-versus-Host Disease Signaling	3.76
ILK Signaling	4.26	Protein Kinase A Signaling	3.76
Phagosome Formation	4.24	Glioma Invasiveness Signaling	3.74
Virus Entry via Endocytic Pathways	4.22	IL-6 Signaling	3.72
Antigen Presentation Pathway	4.19	PCP pathway	3.7
PI3K Signaling in B Lymphocytes	4.17	Epithelial Adherens Junction Signaling	3.64
Caveolar-mediated Endocytosis Signaling	3.94	Pathogenesis of Multiple Sclerosis	3.63

Role of JAK1 and JAK3 in γc Cytokine Signaling	3.94	Production of Nitric Oxide and Reactive Oxygen Species in Macrophages	3.56
P2Y Purigenic Receptor Signaling Pathway	3.89	Thrombin Signaling	3.54
Glioblastoma Multiforme Signaling	3.87	iCOS-iCOSL Signaling in T Helper Cells	3.54
Interferon Signaling	3.86	Glioblastoma Multiforme Signaling	3.46
Role of NANOG in Mammalian Embryonic Stem Cell Pluripotency	3.85	Calcium Signaling	3.43
HMGB1 Signaling	3.82	Adipogenesis pathway	3.38
TGF-β Signaling	3.81	Relaxin Signaling	3.37
Factors Promoting Cardiogenesis in Vertebrates	3.8	Induction of Apoptosis by HIV1	3.36
Antioxidant Action of Vitamin C	3.73	IL-10 Signaling	3.34
G Beta Gamma Signaling	3.72	Agrin Interactions at Neuromuscular Junction	3.27
Germ Cell-Sertoli Cell Junction Signaling	3.69	Germ Cell-Sertoli Cell Junction Signaling	3.26
FAK Signaling	3.64	Virus Entry via Endocytic Pathways	3.25
Integrin Signaling	3.63	Autoimmune Thyroid Disease Signaling	3.23
Breast Cancer Regulation by Stathmin1	3.58	TGF-β Signaling	3.15
Glioma Invasiveness Signaling	3.57	Primary Immunodeficiency Signaling	3.15
Role of NFAT in Cardiac Hypertrophy	3.56	Chemokine Signaling	3.14
IL-6 Signaling	3.51	Extrinsic Prothrombin Activation Pathway	3.13
Macropinocytosis Signaling	3.47	Role of NANOG in Mammalian Embryonic Stem Cell Pluripotency	3.12
Ovarian Cancer Signaling	3.46	Clathrin-mediated Endocytosis Signaling	3.07
Cellular Effects of Sildenafil (Viagra)	3.32	B Cell Development	3.05
Fcγ Receptor-mediated Phagocytosis in Macrophages and Monocytes	3.32	Apoptosis Signaling	3.04

IL-10 Signaling	3.31	Regulation of Cellular Mechanics by Calpain Protease	3.03
CCR5 Signaling in Macrophages	3.22	3-phosphoinositide Biosynthesis	3
Type I Diabetes Mellitus Signaling	3.22	Integrin Signaling	2.98
Actin Cytoskeleton Signaling	3.2	Calcium-induced T Lymphocyte Apoptosis	2.95
fMLP Signaling in Neutrophils	3.2	p38 MAPK Signaling	2.94
GPCR-Mediated Nutrient Sensing in Enteroendocrine Cells	3.14	Prolactin Signaling	2.91
Role of Tissue Factor in Cancer	3.14	Gαs Signaling	2.9
Antiproliferative Role of Somatostatin Receptor 2	3.13	MSP-RON Signaling Pathway	2.89
Calcium Signaling	3.12	Factors Promoting Cardiogenesis in Vertebrates	2.88
Sphingosine-1-phosphate Signaling	3.08	cAMP-mediated signaling	2.86
Aryl Hydrocarbon Receptor Signaling	3.05	Fcγ Receptor-mediated Phagocytosis in Macrophages and Monocytes	2.83
Pancreatic Adenocarcinoma Signaling	3.05	IL-9 Signaling	2.8
Chemokine Signaling	3.04	Antioxidant Action of Vitamin C	2.77
Wnt/β-catenin Signaling	3	CCR5 Signaling in Macrophages	2.77
PTEN Signaling	2.99	Role of Tissue Factor in Cancer	2.72
Gα12/13 Signaling	2.98	Paxillin Signaling	2.72
Regulation of Cellular Mechanics by Calpain Protease	2.97	Wnt/β-catenin Signaling	2.71
Phospholipases	2.96	Gαq Signaling	2.69
Serotonin Receptor Signaling	2.95	Cytotoxic T Lymphocyte-mediated Apoptosis of Target Cells	2.68
Gap Junction Signaling	2.95	Sphingosine-1-phosphate Signaling	2.68
Renin-Angiotensin Signaling	2.93	Mouse Embryonic Stem Cell Pluripotency	2.63

HER-2 Signaling in Breast Cancer	2.92	Role of MAPK Signaling in the Pathogenesis of Influenza	2.59
FXR/RXR Activation	2.9	Ovarian Cancer Signaling	2.58
PKCθ Signaling in T Lymphocytes	2.87	Leukotriene Biosynthesis	2.57
MSP-RON Signaling Pathway	2.79	FXR/RXR Activation	2.56
Mouse Embryonic Stem Cell Pluripotency	2.78	CTLA4 Signaling in Cytotoxic T Lymphocytes	2.54
Cholecystokinin/Gastrin-mediated Signaling	2.76	Ephrin Receptor Signaling	2.54
IL-9 Signaling	2.73	STAT3 Pathway	2.54
Epithelial Adherens Junction Signaling	2.72	Wnt/Ca+ pathway	2.51
IL-1 Signaling	2.71	Cardiac Hypertrophy Signaling	2.5
Acute Myeloid Leukemia Signaling	2.71	Glucocorticoid Receptor Signaling	2.49
nNOS Signaling in Skeletal Muscle Cells	2.7	Protein Citrullination	2.42
Gustation Pathway	2.69	nNOS Signaling in Skeletal Muscle Cells	2.42
Production of Nitric Oxide and Reactive Oxygen Species in Macrophages	2.68	Actin Cytoskeleton Signaling	2.42
Bladder Cancer Signaling	2.62	PPAR Signaling	2.41
p70S6K Signaling	2.62	Role of Hypercytokinemia/hyperchemokinemia in the Pathogenesis of Influenza	2.39
PPAR Signaling	2.58	Nur77 Signaling in T Lymphocytes	2.38
Eicosanoid Signaling	2.55	Gap Junction Signaling	2.35
Autoimmune Thyroid Disease Signaling	2.54	G-Protein Coupled Receptor Signaling	2.35
Prolactin Signaling	2.53	D-myo-inositol-5-phosphate Metabolism	2.33
Cardiomyocyte Differentiation via BMP Receptors	2.51	Taurine Biosynthesis	2.25
iCOS-iCOSL Signaling in T Helper Cells	2.5	Phospholipases	2.22
RhoA Signaling	2.5	CXCR4 Signaling	2.2

Apoptosis Signaling	2.49	Pancreatic Adenocarcinoma Signaling	2.14
ERK5 Signaling	2.46	Growth Hormone Signaling	2.14
Semaphorin Signaling in Neurons	2.44	Inhibition of Matrix Metalloproteases	2.12
PAK Signaling	2.42	Hematopoiesis from Pluripotent Stem Cells	2.12
Oncostatin M Signaling	2.4	IL-2 Signaling	2.11
Netrin Signaling	2.37	Acute Myeloid Leukemia Signaling	2.1
Hepatic Cholestasis	2.37	Ephrin B Signaling	2.1
Regulation of Actin-based Motility by Rho	2.36	Renin-Angiotensin Signaling	2.07
CD28 Signaling in T Helper Cells	2.32	Cholecystokinin/Gastrin-mediated Signaling	2.07
Reelin Signaling in Neurons	2.3	Reelin Signaling in Neurons	2.06
Death Receptor Signaling	2.3	Aryl Hydrocarbon Receptor Signaling	2.05
B Cell Development	2.29	IL-3 Signaling	2.05
T Cell Receptor Signaling	2.28	Role of PKR in Interferon Induction and Antiviral Response	2.05
BMP signaling pathway	2.28	Role of JAK family kinases in IL-6-type Cytokine Signaling	2.04
Superpathway of Inositol Phosphate Compounds	2.25	P2Y Purigenic Receptor Signaling Pathway	2
Role of MAPK Signaling in the Pathogenesis of Influenza	2.19	IL-15 Signaling	1.96
GPCR-Mediated Integration of Enteroendocrine Signaling Exemplified by an L Cell	2.19	Gustation Pathway	1.94
IL-3 Signaling	2.18	NF-κB Activation by Viruses	1.93
JAK/Stat Signaling	2.18	Role of JAK2 in Hormone-like Cytokine Signaling	1.92
Xenobiotic Metabolism Signaling	2.14	Bladder Cancer Signaling	1.89
CREB Signaling in Neurons	2.08	VDR/RXR Activation	1.87
Spermine and Spermidine Degradation I	2.05	ErbB2-ErbB3 Signaling	1.87

Melatonin Degradation II	2.05	Tight Junction Signaling	1.85
Clathrin-mediated Endocytosis Signaling	2.04	HER-2 Signaling in Breast Cancer	1.85
Cardiac β-adrenergic Signaling	2.04	Cardiomyocyte Differentiation via BMP Receptors	1.84
Neuropathic Pain Signaling In Dorsal Horn Neurons	2.03	Regulation of IL-2 Expression in Activated and Anergic T Lymphocytes	1.83
FcγRIIB Signaling in B Lymphocytes	2.03	Sertoli Cell-Sertoli Cell Junction Signaling	1.83
GADD45 Signaling	2.02	FAK Signaling	1.79
Graft-versus-Host Disease Signaling	2.01	FcγRIIB Signaling in B Lymphocytes	1.78
NF-κB Activation by Viruses	2	Inflammasome pathway	1.75
Notch Signaling	2	Macropinocytosis Signaling	1.75
ErbB Signaling	1.97	Role of NFAT in Cardiac Hypertrophy	1.74
ERK/MAPK Signaling	1.97	ERK5 Signaling	1.73
Estrogen-mediated S-phase Entry	1.96	TNFR2 Signaling	1.72
Ceramide Signaling	1.93	GM-CSF Signaling	1.7
Intrinsic Prothrombin Activation Pathway	1.93	Triacylglycerol Biosynthesis	1.69
Ephrin A Signaling	1.91	UVA-Induced MAPK Signaling	1.69
CDK5 Signaling	1.91	JAK/Stat Signaling	1.68
Amyotrophic Lateral Sclerosis Signaling	1.89	D-myo-inositol (1,4,5,6)-Tetrakisphosphate Biosynthesis	1.66
GNRH Signaling	1.88	D-myo-inositol (3,4,5,6)-tetrakisphosphate Biosynthesis	1.66
Estrogen-Dependent Breast Cancer Signaling	1.88	Thrombopoietin Signaling	1.64
Role of JAK family kinases in IL-6-type Cytokine Signaling	1.84	Allograft Rejection Signaling	1.64
IL-4 Signaling	1.84	Actin Nucleation by ARP-WASP Complex	1.64
CCR3 Signaling in Eosinophils	1.83	Netrin Signaling	1.61
Role of PKR in Interferon Induction and Antiviral Response	1.83	Prostanoid Biosynthesis	1.6
Retinol Biosynthesis	1.83	Sperm Motility	1.59

B Cell Receptor Signaling	1.82	IL-12 Signaling and Production in Macrophages	1.58
VDR/RXR Activation	1.82	ERK/MAPK Signaling	1.58
Coagulation System	1.82	PPARα/RXRα Activation	1.57
Actin Nucleation by ARP-WASP Complex	1.81	3-phosphoinositide Degradation	1.57
Triacylglycerol Biosynthesis	1.77	Sumoylation Pathway	1.56
3-phosphoinositide Biosynthesis	1.76	Dermatan Sulfate Degradation (Metazoa)	1.55
Taurine Biosynthesis	1.74	Estrogen-Dependent Breast Cancer Signaling	1.54
L-cysteine Degradation III	1.74	Type II Diabetes Mellitus Signaling	1.54
Tyrosine Degradation I	1.7	Hepatic Cholestasis	1.52
ErbB2-ErbB3 Signaling	1.69	Uracil Degradation II (Reductive)	1.51
IL-12 Signaling and Production in Macrophages	1.68	Thymine Degradation	1.51
Growth Hormone Signaling	1.66	G Beta Gamma Signaling	1.5
G Protein Signaling Mediated by Tubby	1.64	Systemic Lupus Erythematosus Signaling	1.49
Telomerase Signaling	1.63	Fc Epsilon RI Signaling	1.47
IL-15 Signaling	1.62	PTEN Signaling	1.47
Pathogenesis of Multiple Sclerosis	1.62	Calcium Transport I	1.47
Pyridoxal 5'-phosphate Salvage Pathway	1.6	Glycine Betaine Degradation	1.47
Thrombopoietin Signaling	1.6	OX40 Signaling Pathway	1.46
14-3-3-mediated Signaling	1.59	IL-4 Signaling	1.46
April Mediated Signaling	1.57	Cellular Effects of Sildenafil (Viagra)	1.46
Angiopoietin Signaling	1.57	Gα12/13 Signaling	1.46
Induction of Apoptosis by HIV1	1.56	Gαi Signaling	1.45
TNFR1 Signaling	1.56	PDGF Signaling	1.43
Putrescine Degradation III	1.55	fMLP Signaling in Neutrophils	1.42
Fc Epsilon RI Signaling	1.55	B Cell Receptor Signaling	1.41
Sperm Motility	1.55	TWEAK Signaling	1.41

tRNA Splicing	1.5	Regulation of Actin-based Motility by Rho	1.4
UVA-Induced MAPK Signaling	1.5	IL-1 Signaling	1.4
GABA Receptor Signaling	1.49	Role of RIG1-like Receptors in Antiviral Innate Immunity	1.4
Role of JAK2 in Hormone-like Cytokine Signaling	1.48	Serotonin Receptor Signaling	1.4
GM-CSF Signaling	1.48	Ceramide Signaling	1.34
Erythropoietin Signaling	1.47	IL-15 Production	1.32
Leptin Signaling in Obesity	1.46	Acetone Degradation I (to Methylglyoxal)	1.32
TNFR2 Signaling	1.46	Tyrosine Degradation I	1.31
Calcium Transport I	1.45	Cardiac β-adrenergic Signaling	1.28
Role of JAK1, JAK2 and TYK2 in Interferon Signaling	1.45	GPCR-Mediated Nutrient Sensing in Enteroendocrine Cells	1.27
UDP-N-acetyl-D-glucosamine Biosynthesis II	1.44	Antiproliferative Role of Somatostatin Receptor 2	1.27
Sertoli Cell-Sertoli Cell Junction Signaling	1.43	IGF-1 Signaling	1.26
Neuroprotective Role of THOP1 in Alzheimer's Disease	1.43	GNRH Signaling	1.22
PPARα/RXRα Activation	1.43	Xenobiotic Metabolism Signaling	1.19
Chronic Myeloid Leukemia Signaling	1.42	p70S6K Signaling	1.17
TR/RXR Activation	1.42	CDK5 Signaling	1.17
CTLA4 Signaling in Cytotoxic T Lymphocytes	1.38	Chondroitin and Dermatan Biosynthesis	1.16
α-Adrenergic Signaling	1.38	Serotonin and Melatonin Biosynthesis	1.16
IL-2 Signaling	1.35	tRNA Splicing	1.16
Neurotrophin/TRK Signaling	1.33	Estrogen Biosynthesis	1.16
Neuregulin Signaling	1.33	Glutathione-mediated Detoxification	1.15
Nur77 Signaling in T Lymphocytes	1.3	Corticotropin Releasing Hormone Signaling	1.13

Thiosulfate Disproportionation III (Rhodanese)	1.3	Telomerase Signaling	1.13
Primary Immunodeficiency Signaling	1.28	Natural Killer Cell Signaling	1.13
RAR Activation	1.27	RhoA Signaling	1.13
Inflammasome pathway	1.26	L-dopachrome Biosynthesis	1.12
PDGF Signaling	1.25	Sorbitol Degradation I	1.12
Type II Diabetes Mellitus Signaling	1.25	Asparagine Biosynthesis I	1.12
PEDF Signaling	1.25	4-hydroxybenzoate Biosynthesis	1.12
HIF1α Signaling	1.24	4-hydroxyphenylpyruvate Biosynthesis	1.12
Natural Killer Cell Signaling	1.21	RANK Signaling in Osteoclasts	1.12
D-myo-inositol-5-phosphate Metabolism	1.19	Thyroid Cancer Signaling	1.11
p38 MAPK Signaling	1.17	Cdc42 Signaling	1.11
LPS-stimulated MAPK Signaling	1.17	Chondroitin Sulfate Biosynthesis (Late Stages)	1.1
Renal Cell Carcinoma Signaling	1.12	Ephrin A Signaling	1.1
IGF-1 Signaling	1.12	MIF Regulation of Innate Immunity	1.07
CNTF Signaling	1.11	G Protein Signaling Mediated by Tubby	1.05
Airway Pathology in Chronic Obstructive Pulmonary Disease	1.09	Putrescine Degradation III	1.05
Thyroid Cancer Signaling	1.08	HGF Signaling	1.04
GDNF Family Ligand-Receptor Interactions	1.08	Activation of IRF by Cytosolic Pattern Recognition Receptors	1.03
B Cell Activating Factor Signaling	1.08	Huntington's Disease Signaling	1.01
Role of Wnt/GSK-3β Signaling in the Pathogenesis of Influenza	1.08	Chondroitin Sulfate Degradation (Metazoa)	1.01
Myc Mediated Apoptosis Signaling	1.07	MIF-mediated Glucocorticoid Regulation	1.01
RANK Signaling in Osteoclasts	1.07	Salvage Pathways of Pyrimidine Ribonucleotides	0.998

Salvage Pathways of Pyrimidine Ribonucleotides	1.06	IL-22 Signaling	0.992
Choline Biosynthesis III	1.06	Tumoricidal Function of Hepatic Natural Killer Cells	0.992
Melanocyte Development and Pigmentation Signaling	1.06	Role of JAK1, JAK2 and TYK2 in Interferon Signaling	0.992
Uracil Degradation II (Reductive)	1.04	Glutathione Redox Reactions I	0.992
Ascorbate Recycling (Cytosolic)	1.04	Leptin Signaling in Obesity	0.979
Thymine Degradation	1.04	iNOS Signaling	0.958
L-cysteine Degradation I	1.04	Pyridoxal 5'-phosphate Salvage Pathway	0.944
Non-Small Cell Lung Cancer Signaling	1.04	Granzyme B Signaling	0.942
Dopamine Receptor Signaling	1.04	γ-glutamyl Cycle	0.942
Tight Junction Signaling	1.03	IL-17A Signaling in Gastric Cells	0.942
HGF Signaling	1.03	Bupropion Degradation	0.942
Glucocorticoid Receptor Signaling	1.02	Tryptophan Degradation X (Mammalian, via Tryptamine)	0.942
Glutathione Redox Reactions I	1.02	Role of Wnt/GSK-3β Signaling in the Pathogenesis of Influenza	0.934
CDP-diacylglycerol Biosynthesis I	1.02	GDNF Family Ligand-Receptor Interactions	0.934
CD40 Signaling	1	Neurotrophin/TRK Signaling	0.934
Huntington's Disease Signaling	1	BMP signaling pathway	0.934
Glutathione-mediated Detoxification	0.992	Airway Pathology in Chronic Obstructive Pulmonary Disease	0.932
Glioma Signaling	0.988	Dopamine Degradation	0.923
Calcium-induced T Lymphocyte Apoptosis	0.984	Angiopoietin Signaling	0.908
UVC-Induced MAPK Signaling	0.972	Neuregulin Signaling	0.905
Rac Signaling	0.971	Phagosome Maturation	0.903
Regulation of IL-2 Expression in Activated and Anergic T Lymphocytes	0.964	Amyotrophic Lateral Sclerosis Signaling	0.886

Leukotriene Biosynthesis	0.958	Breast Cancer Regulation by Stathmin1	0.883
Phenylalanine Degradation IV (Mammalian, via Side Chain)	0.958	PAK Signaling	0.861
Corticotropin Releasing Hormone Signaling	0.958	Erythropoietin Signaling	0.859
Androgen Signaling	0.958	D-myo-inositol (1,4,5)-Trisphosphate Biosynthesis	0.85
Prostanoid Biosynthesis	0.954	Nitric Oxide Signaling in the Cardiovascular System	0.846
IL-17A Signaling in Gastric Cells	0.949	Chondroitin Sulfate Biosynthesis	0.842
Tryptophan Degradation X (Mammalian, via Tryptamine)	0.949	L-cysteine Degradation III	0.839
Melanoma Signaling	0.93	4-hydroxyproline Degradation I	0.839
Role of Hypercytokinemia/hyperchemokinemia in the Pathogenesis of Influenza	0.923	Anandamide Degradation	0.839
Antiproliferative Role of TOB in T Cell Signaling	0.885	Remodeling of Epithelial Adherens Junctions	0.836
Phosphatidylglycerol Biosynthesis II (Non-plastidic)	0.885	Differential Regulation of Cytokine Production in Macrophages and T Helper Cells by IL-17A and IL-17F	0.826
iNOS Signaling	0.876	April Mediated Signaling	0.812
Cytotoxic T Lymphocyte-mediated Apoptosis of Target Cells	0.876	Notch Signaling	0.812
Chondroitin Sulfate Degradation (Metazoa)	0.87	Chronic Myeloid Leukemia Signaling	0.8
Vitamin-C Transport	0.87	SAPK/JNK Signaling	0.8
Acetyl-CoA Biosynthesis III (from Citrate)	0.868	TNFR1 Signaling	0.795
Melatonin Degradation III	0.868	Dopamine-DARPP32 Feedback in cAMP Signaling	0.794
Sorbitol Degradation I	0.868	Dermatan Sulfate Biosynthesis	0.788
Asparagine Biosynthesis I	0.868	GADD45 Signaling	0.775
Citrulline Degradation	0.868	Histamine Degradation	0.775

4-hydroxybenzoate Biosynthesis	0.868	GPCR-Mediated Integration of Enteroendocrine Signaling Exemplified by an L Cell	0.763
4-hydroxyphenylpyruvate Biosynthesis	0.868	Retinoic acid Mediated Apoptosis Signaling	0.762
Protein Citrullination	0.858	B Cell Activating Factor Signaling	0.746
Citrulline-Nitric Oxide Cycle	0.858	Neuroprotective Role of THOP1 in Alzheimer's Disease	0.746
Pregnenolone Biosynthesis	0.843	Transcriptional Regulatory Network in Embryonic Stem Cells	0.746
Maturity Onset Diabetes of Young (MODY) Signaling	0.838	Noradrenaline and Adrenaline Degradation	0.746
eNOS Signaling	0.838	PEDF Signaling	0.746
IL-15 Production	0.825	14-3-3-mediated Signaling	0.745
D-myo-inositol (1,4,5)-Trisphosphate Biosynthesis	0.825	Sonic Hedgehog Signaling	0.731
Endometrial Cancer Signaling	0.823	Granzyme A Signaling	0.728
Cell Cycle: G1/S Checkpoint Regulation	0.823	CD27 Signaling in Lymphocytes	0.711
D-myo-inositol (1,4,5,6)-Tetrakisphosphate Biosynthesis	0.807	Docosahexaenoic Acid (DHA) Signaling	0.711
D-myo-inositol (3,4,5,6)-tetrakisphosphate Biosynthesis	0.807	LPS-stimulated MAPK Signaling	0.704
Melatonin Signaling	0.801	TR/RXR Activation	0.695
Dermatan Sulfate Degradation (Metazoa)	0.791	Endometrial Cancer Signaling	0.689
γ-glutamyl Cycle	0.791	Glioma Signaling	0.689
Role of Oct4 in Mammalian Embryonic Stem Cell Pluripotency	0.789	UVC-Induced MAPK Signaling	0.686
SAPK/JNK Signaling	0.788	Semaphorin Signaling in Neurons	0.685
Phagosome Maturation	0.784	Oxidative Ethanol Degradation III	0.684
Cyclins and Cell Cycle Regulation	0.784	L-carnitine Biosynthesis	0.679
Noradrenaline and Adrenaline Degradation	0.78	D-glucuronate Degradation I	0.679

TWEAK Signaling	0.773	Methionine Salvage II (Mammalian)	0.679
FLT3 Signaling in Hematopoietic Progenitor Cells	0.769	Thiosulfate Disproportionation III (Rhodanese)	0.679
NRF2-mediated Oxidative Stress Response	0.75	Heparan Sulfate Biosynthesis (Late Stages)	0.675
nNOS Signaling in Neurons	0.748	Androgen Signaling	0.671
Stearate Biosynthesis I (Animals)	0.737	VEGF Family Ligand-Receptor Interactions	0.665
Dopamine Degradation	0.726	CREB Signaling in Neurons	0.652
Arginine Biosynthesis IV	0.721	Hematopoiesis from Multipotent Stem Cells	0.644
Proline Biosynthesis II (from Arginine)	0.721	Polyamine Regulation in Colon Cancer	0.643
Urea Cycle	0.721	Fatty Acid α-oxidation	0.643
Serotonin and Melatonin Biosynthesis	0.721	Lymphotoxin β Receptor Signaling	0.623
Glycine Cleavage Complex	0.721	Differential Regulation of Cytokine Production in Intestinal Epithelial Cells by IL-17A and IL-17F	0.606
Role of IL-17A in Arthritis	0.721	Choline Biosynthesis III	0.592
Ubiquinol-10 Biosynthesis (Eukaryotic)	0.721	RAR Activation	0.579
γ-linolenate Biosynthesis II (Animals)	0.721	Airway Inflammation in Asthma	0.571
Mitochondrial L-carnitine Shuttle Pathway	0.721	Spermine and Spermidine Degradation I	0.571
Lymphotoxin β Receptor Signaling	0.721	Catecholamine Biosynthesis	0.571
Synaptic Long Term Potentiation	0.72	Phenylethylamine Degradation I	0.571
Prostate Cancer Signaling	0.702	Melatonin Degradation II	0.571
Dopamine-DARPP32 Feedback in cAMP Signaling	0.69	L-cysteine Degradation I	0.571
Activation of IRF by Cytosolic Pattern Recognition Receptors	0.673	Fatty Acid β-oxidation III (Unsaturated, Odd Number)	0.571
Acyl-CoA Hydrolysis	0.667	CDP-diacylglycerol Biosynthesis I	0.571

Tumoricidal Function of Hepatic Natural Killer Cells	0.661	Myc Mediated Apoptosis Signaling	0.564
IL-22 Signaling	0.661	nNOS Signaling in Neurons	0.556
Remodeling of Epithelial Adherens Junctions	0.66	Dermatan Sulfate Biosynthesis (Late Stages)	0.556
Role of RIG1-like Receptors in Antiviral Innate Immunity	0.657	Melanocyte Development and Pigmentation Signaling	0.544
3-phosphoinositide Degradation	0.655	Heparan Sulfate Biosynthesis	0.544
Sumoylation Pathway	0.651	Ethanol Degradation IV	0.538
Chondroitin Sulfate Biosynthesis (Late Stages)	0.638	Synaptic Long Term Potentiation	0.534
p53 Signaling	0.617	autophagy	0.528
Small Cell Lung Cancer Signaling	0.615	FLT3 Signaling in Hematopoietic Progenitor Cells	0.511
Allograft Rejection Signaling	0.615	Lipid Antigen Presentation by CD1	0.507
Ethanol Degradation IV	0.612	Phosphatidylglycerol Biosynthesis II (Non-plastidic)	0.507
4-hydroxyproline Degradation I	0.597	ErbB Signaling	0.499
Anandamide Degradation	0.597	Trehalose Degradation II (Trehalase)	0.49
Glycine Degradation (Creatine Biosynthesis)	0.597	Eumelanin Biosynthesis	0.49
GDP-L-fucose Biosynthesis I (from GDP-D-mannose)	0.597	Citrulline-Nitric Oxide Cycle	0.49
Formaldehyde Oxidation II (Glutathione-dependent)	0.597	α-Adrenergic Signaling	0.48
Histidine Degradation VI	0.596	CNTF Signaling	0.473
IL-17 Signaling	0.59	RAN Signaling	0.466
AMPK Signaling	0.579	Role of PI3K/AKT Signaling in the Pathogenesis of Influenza	0.46
Docosahexaenoic Acid (DHA) Signaling	0.573	Cell Cycle: G1/S Checkpoint Regulation	0.455
UVB-Induced MAPK Signaling	0.557	Neuropathic Pain Signaling In Dorsal Horn Neurons	0.45
Hereditary Breast Cancer Signaling	0.549	Retinol Biosynthesis	0.45

The Visual Cycle	0.549	PXR/RXR Activation	0.439
MIF-mediated Glucocorticoid Regulation	0.545	FGF Signaling	0.437
DNA Double-Strand Break Repair by Homologous Recombination	0.533	Triacylglycerol Degradation	0.433
Superpathway of Citrulline Metabolism	0.533	Aldosterone Signaling in Epithelial Cells	0.433
Transcriptional Regulatory Network in Embryonic Stem Cells	0.531	Mitochondrial L-carnitine Shuttle Pathway	0.432
Superoxide Radicals Degradation	0.527	VEGF Signaling	0.43
Hematopoiesis from Pluripotent Stem Cells	0.522	CD40 Signaling	0.43
VEGF Family Ligand-Receptor Interactions	0.521	Insulin Receptor Signaling	0.429
Retinoate Biosynthesis I	0.509	Stearate Biosynthesis I (Animals)	0.429
EGF Signaling	0.506	Arginine Biosynthesis IV	0.426
MIF Regulation of Innate Immunity	0.499	Urea Cycle	0.426
Cdc42 Signaling	0.485	UDP-N-acetyl-D-glucosamine Biosynthesis II	0.426
FGF Signaling	0.478	Zymosterol Biosynthesis	0.426
Systemic Lupus Erythematosus Signaling	0.469	GDP-mannose Biosynthesis	0.426
Cancer Drug Resistance By Drug Efflux	0.464	UVB-Induced MAPK Signaling	0.423
Fatty Acid α-oxidation	0.459	NRF2-mediated Oxidative Stress Response	0.419
L-carnitine Biosynthesis	0.451	eNOS Signaling	0.416
NADH Repair	0.451	Role of Cytokines in Mediating Communication between Immune Cells	0.416
Coenzyme A Biosynthesis	0.451	Rac Signaling	0.414
Thyronamine and Iodothyronamine Metabolism	0.451	CCR3 Signaling in Eosinophils	0.41
Biotin-carboxyl Carrier Protein Assembly	0.451	Superpathway of Melatonin Degradation	0.407

Thyroid Hormone Metabolism I (via Deiodination)	0.451	Melanoma Signaling	0.399
Glutamate Degradation II	0.451	EGF Signaling	0.392
S-adenosyl-L-methionine Biosynthesis	0.451	Prostate Cancer Signaling	0.384
Tyrosine Biosynthesis IV	0.451	4-1BB Signaling in T Lymphocytes	0.381
Aspartate Biosynthesis	0.451	1D-myo-inositol Hexakisphosphate Biosynthesis II (Mammalian)	0.372
Nitric Oxide Signaling in the Cardiovascular System	0.443	D-myo-inositol (1,3,4)-trisphosphate Biosynthesis	0.372
Role of p14/p19ARF in Tumor Suppression	0.44	Role of IL-17F in Allergic Inflammatory Airway Diseases	0.372
Glutamate Receptor Signaling	0.437	Melatonin Signaling	0.35
Extrinsic Prothrombin Activation Pathway	0.43	DNA Methylation and Transcriptional Repression Signaling	0.346
Parkinson's Signaling	0.43	The Visual Cycle	0.346
Sonic Hedgehog Signaling	0.415	Role of Oct4 in Mammalian Embryonic Stem Cell Pluripotency	0.338
Chondroitin Sulfate Biosynthesis	0.413	IL-17 Signaling	0.338
Amyloid Processing	0.413	Inositol Pyrophosphates Biosynthesis	0.332
Aldosterone Signaling in Epithelial Cells	0.399	Tryptophan Degradation to 2-amino-3-carboxymuconate Semialdehyde	0.332
Glycine Betaine Degradation	0.396	Maturity Onset Diabetes of Young (MODY) Signaling	0.322
Methionine Degradation I (to Homocysteine)	0.387	Endoplasmic Reticulum Stress Pathway	0.322
VEGF Signaling	0.372	Retinoate Biosynthesis I	0.322
Triacylglycerol Degradation	0.366	IL-17A Signaling in Fibroblasts	0.304
OX40 Signaling Pathway	0.365	HIF1α Signaling	0.3
Superpathway of Methionine Degradation	0.355	Melatonin Degradation I	0.297
Airway Inflammation in Asthma	0.355	GDP-glucose Biosynthesis	0.296

Arsenate Detoxification I (Glutaredoxin)	0.355	Nicotine Degradation II	0.285
Retinoate Biosynthesis II	0.355	NGF Signaling	0.282
Heme Degradation	0.355	Non-Small Cell Lung Cancer Signaling	0.279
Catecholamine Biosynthesis	0.355	Dopamine Receptor Signaling	0.279
Phenylethylamine Degradation I	0.355	Ethanol Degradation II	0.272
Proline Biosynthesis I	0.355	Embryonic Stem Cell Differentiation into Cardiac Lineages	0.265
N-acetylglucosamine Degradation II	0.355	Glucose and Glucose-1-phosphate Degradation	0.265
Phenylalanine Degradation I (Aerobic)	0.355	Estrogen-mediated S-phase Entry	0.261
Fatty Acid β-oxidation III (Unsaturated, Odd Number)	0.355	Superpathway of D-myo-inositol (1,4,5)-trisphosphate Metabolism	0.261
Differential Regulation of Cytokine Production in Macrophages and T Helper Cells by IL-17A and IL-17F	0.348	Tryptophan Degradation III (Eukaryotic)	0.261
Insulin Receptor Signaling	0.347	Cell Cycle Control of Chromosomal Replication	0.257
Mineralocorticoid Biosynthesis	0.346	Mineralocorticoid Biosynthesis	0.239
Role of Cytokines in Mediating Communication between Immune Cells	0.345	UDP-N-acetyl-D-galactosamine Biosynthesis II	0.239
Dermatan Sulfate Biosynthesis (Late Stages)	0.34	Antiproliferative Role of TOB in T Cell Signaling	0.227
Role of PI3K/AKT Signaling in the Pathogenesis of Influenza	0.34	NAD Salvage Pathway II	0.227
Glycolysis I	0.322	Glycolysis I	0.227
Histamine Degradation	0.314	Gluconeogenesis I	0.227
DNA damage-induced 14-3-3σ Signaling	0.314	Glucocorticoid Biosynthesis	0.215
Cysteine Biosynthesis III (mammalia)	0.314	Role of IL-17A in Psoriasis	0.195

Inhibition of Angiogenesis by TSP1	0.304	Fatty Acid Activation	0.195
Hematopoiesis from Multipotent Stem Cells	0.303	Cholesterol Biosynthesis I	0.195
Glucocorticoid Biosynthesis	0.303	NAD Phosphorylation and Dephosphorylation	0.195
Cell Cycle: G2/M DNA Damage Checkpoint Regulation	0.299	Bile Acid Biosynthesis, Neutral Pathway	0.195
Acetone Degradation I (to Methylglyoxal)	0.295	Cholesterol Biosynthesis II (via 24,25-dihydrolanosterol)	0.195
Creatine-phosphate Biosynthesis	0.286	Guanosine Nucleotides Degradation III	0.195
CMP-N-acetylneuraminate Biosynthesis I (Eukaryotes)	0.286	Cholesterol Biosynthesis III (via Desmosterol)	0.195
Lysine Degradation V	0.286		
ErbB4 Signaling	0.285		
DNA Methylation and Transcriptional Repression Signaling	0.283		
IL-17A Signaling in Fibroblasts	0.281		
NGF Signaling	0.28		
PXR/RXR Activation	0.278		
Role of IL-17A in Psoriasis	0.266		
Fatty Acid Activation	0.266		
Oleate Biosynthesis II (Animals)	0.266		
Oxidative Ethanol Degradation III	0.255		
Chondroitin and Dermatan Biosynthesis	0.235		
Arginine Degradation VI (Arginase 2 Pathway)	0.235		
Thioredoxin Pathway	0.235		
Pentose Phosphate Pathway (Non-oxidative Branch)	0.235		
Rapoport-Luebering Glycolytic Shunt	0.235		
Androgen Biosynthesis	0.234		

Polyamine Regulation in Colon Cancer	0.23		
Aspartate Degradation II	0.194		

Supplementary Table 5: List of qRT-PCR primers

Gene	Accession no.	Forward Primer	Reverse Primer
BCL2	NM_001002949.1	GGGCCATAAGTGAAGTCAGTAG	GAGACCACAGCTTCGTTTCA
BIRC3	NM_001080725.1	CCTGAGCACTGGAGAGAATTAC	CTGCTCTTTCTTTCTCCTCCTC
C1QB	XM_544507	ACTATAAGGCCACCCAGAAGA	GTTGGTGACTATGTGGTCGAAG
C1R	XM_005637209	GAGGCTGGAAGCTGCATTAT	GTAGTCACGGAACTGGTATTGG
C1S	XM_005637210	CTTCACTCACCTGGACATAGAG	CCCACAGAGTTTCCCTTCTT
C3	XM_005633228	CGAAGAAGGAGGGTATCCTAGA	CACAGACAGATGCAGGTAGTT
C5	XM_014118002	CATCCTGTCTCCCTACAAACTG	TGGTCCAGCACATCCTTAAC
C7	XM_546339	CCTGCGTTCAAGGGAAGAAA	CTTTCCCGTGGTTCTCCAATAC
CASP1	NM_001003125	TCAAGCTTTGTCCTCCTGAAA	TGATGAGAGCCAGACGAGTA
CD55	XM_014115065	GATGTTCCAACCAGGCTACTT	AGCCTGGACGACATTCATATTC
CD59	XM_533156	ACCCGGCCTATCACAAATG	GCAACACCCACAAGACAAAG
CFB	XM_003431676	CAGATGAGGCACACGGAATA	CAAGAGGACCCAGATTGTCTAC
CFD	XM_542213	TCACAACGAGGGAGACTGTT	GCCTTCAGGTGTGGTTTGAT
CFH	XM_536110	CAGAGGGTACTCAGGCTACATA	GGATTAAGAGCCACCCATTCTC
CFI	XM_014109975	GGAGGAAAGGCAGCAGTAAT	GAATCCAACACCCTCCGATATAA
FAS	XM_005636650.1	GGCGCGTATCACACCTTAAT	TAACTGGCAACGTCCTTCTG
GAPDH	NM_001003142.2	AACAGTGACACCCACTCTTC	CGGTTGCTGTAGCCAAATTC
IL18	XM_005619484	TTGAGGATATGCCCGATTCTG	AGTTACTGCCAGACCTCTAGT
IL1B	XM_005630074	CCAAAGTCTACCCAAAGAGGAA	ACCAGTTAGGGTACTGAGAAGA
MYD88	XM_534223	TCCACTTGCCTCTTCATTCC	AGCTACTGGTTCCACAAAGG
NLRC5	XM_014109337	GATCAGCTTGGCAGAGAACA	CTCACACGAAACCAGGTCTATC
NLRP3	XM_014115621	CGACTTCAATGGTGAGGAGAAG	CAGCTCATCCCTCTTAGCTTTC
PYCARD	XM_014114362	CCTCACACAAAGGAGCATCA	CTCTGACAGGACTTTCCCATAC
SERPING1	XM_014120908	CGTGGACTCTTCTACCTTGTTG	AACAGTGGACCCGATGAAAG
SOCS1	XM_005622079.1	CCTGGTTGTTGTAGCAGCTTA	CCCTGGTTTGTGCAAATATACTG
SOCS3	NM_001031631.1	GAAGATCCCTCTGGTGTTGAG	TTTCTCGTAGGAGTCCAGGT
STAT1	XM_843260.4	ATTACTCCAGGCCAAAGGAAG	GGTGGACTTCAGACACAGAAA
STAT3	XM_005624459.2	GAAGGACAACCTGAACCTAGAC	CCCAGCCTTACCAACTGATAG
TLR1	XM_014112329	CCAGGTAGCAAGCAAAGTAGTA	GGATAACGGAGACACGTGAAA
TLR10	XM_005618243	GCCTTCTGCTGCTTCTACTT	TGAGCTGTTCTTGGGTTGTT
TLR2	XM_005628829	TCTGGTTCCTTGCTCACTTTC	GACAGGCTGAGTTTCTCAAGTAT
TLR3	XM_540020	GGTACTTGACCTTGGCCTTAAT	CTGCTCGTCAGTTGTAGGTATTT
TLR7	XM_005641003	TTTCCCAGAGCATACAGCTTAG	GCCTCTGATGGGACAAGTAAA
TLR8	XM_003435448	GCTGCCCGTCTTGGAAATA	GGAGCAACTTCAGGCTAGAAA
TLR9	XM_014121723	CACACACCTGTCACTCAAGTA	AGGGTGATGATGTGGTTGTAG
TNFRSF1A	XM_005637258.2	CAGGGTGAAGAGAGCTATTCTG	GGACAGTCATTGTACAGGTAGG
TNFRSF1B	XM_005617982.2	GCTCTTTCTGGGCCAAATTC	GAGCGCTTTCCCACAATTC
XIAP	NM_001284458.1	ATCACTTGAGGCTCTGGTTTC	AGCTGCTCTTCAGCACTAATC

Supplementary Table 6A: List of antibodies used for immunohistochemistry (IHC)

Antigen	Antibody (company and catalog no.)	Dilution used for IHC
TNFRSF1B	Abcam, ab15563	1:50
FAS	Abcam, ab82419	1:100
C3	Abcam, ab112829	1:100
C9	Biorbyt, orb156182	1:200
NLRP3	Adipogen, AG-20B-0014	1:200
CASP1	Abbiotec, 250570	1:200
BIRC3	Abcam, ab23423	1:500
STAT3	Cell Signaling Technology, 9139	1:250
RELA	Cell Signaling Technology, 4764	1:500

Supplementary Table 6B: List of antibodies used for western blot analysis (WB)

Antigen	Antibody (company and catalog no.)	Dilution used for WB
C3	Abcam, ab112829	1:500
NLRP3	Novus Bio, NB100-41104	1:200
PYCARD	Adipogen, AG-25B-0006	1:500
IL1B	Abcam, ab34837	1:300
CASP1	Abbiotec, 250570	1:200
BIRC3	Abcam, ab23423	1:500
BCL2	Cell Signaling Technology, 2870	1:500
STAT3	Cell Signaling Technology, 9139	1:500
p-STAT3	Cell Signaling Technology, 9131	1: 500
SOCS3	Abcam, ab16030	1: 200
B-ACTIN	Abcam, ab8226, ab8227	1:3000

Supplementary Table 6C: Other antibodies that were tested and did not work in canine tissue.

Antigen	Antibody (company and catalog no.)	Lowest dilution tested for IHC
C5b-9	Novus Biologicals, NBP1-05120	1:100
SOCS3	Abcam, ab16030	1:100
BCL2	Cell Signaling Technology, 2870	1:100
STAT1	Cell Signaling Technology, 9176	1:200
TLR1	Novus Biologicals, NB100-56563	1:100
TLR3	Novus Biologicals, NBP2-24875	1:50
TLR7	Novus Biologicals, NBP2-24906	1:100
TLR8	Novus Biologicals, NBP2-24917	1:100
TLR9	Novus Biologicals, NBP2-24729	1:100
NLRC5	Invitrogen, PA5-21017	1:100
PYCARD	Adipogen, AG-25B-0006	1:500
MYD88	Santa Cruz Biotechnology, sc-11356	1:200

Supplementary Information S7: Complete western blot images, corresponding to figure 4

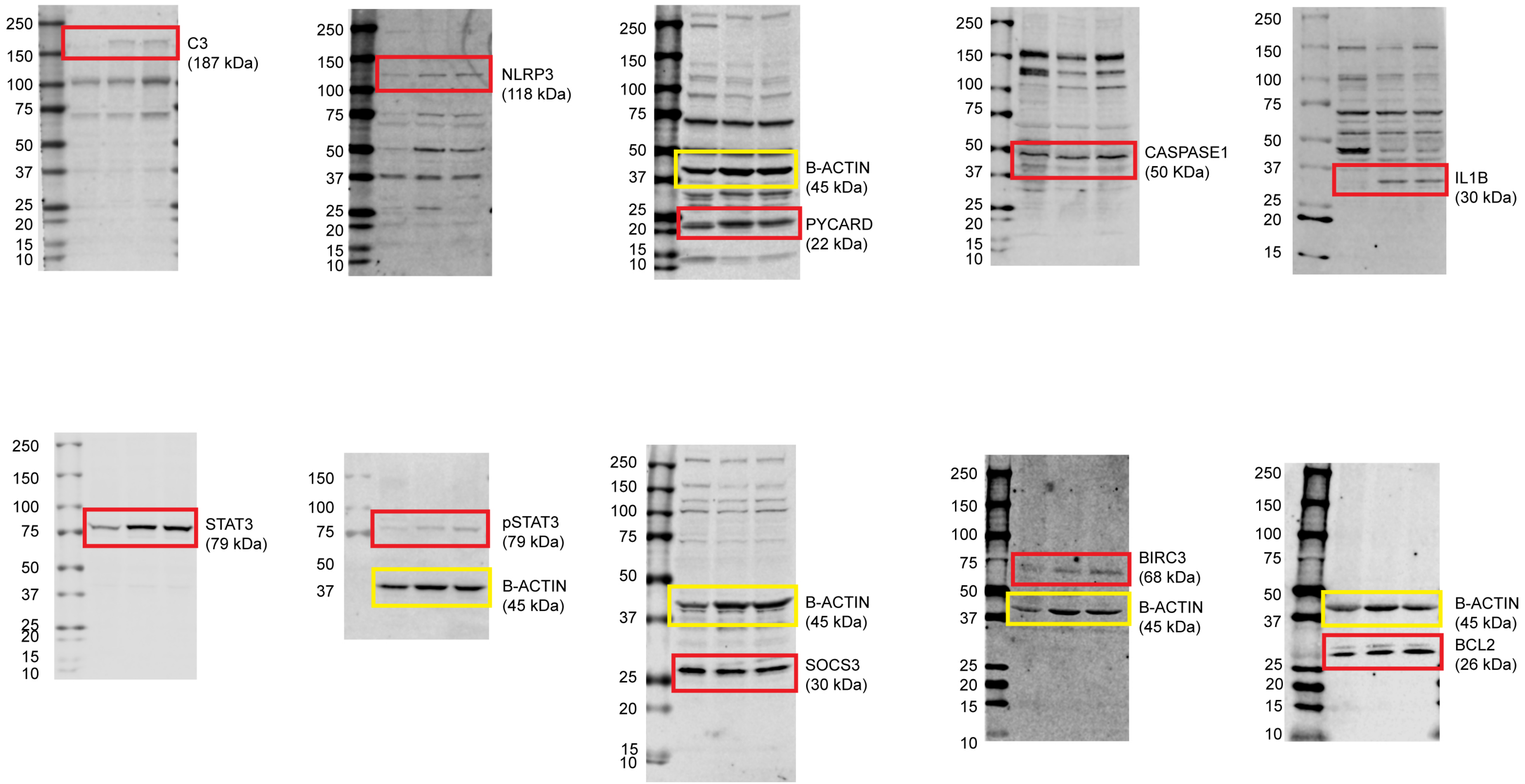

Supplement: Supplementary file 1 — Supplementary Information [file 41598_2017_18236_MOESM1_ESM.pdf]
